# Divalent Multilinking Bonds Control Growth and Morphology of Nanopolymers

**DOI:** 10.1021/acs.nanolett.1c03009

**Published:** 2021-10-14

**Authors:** Yan Xiong, Zhiwei Lin, Deniz Mostarac, Brian Minevich, Qiuyuan Peng, Guolong Zhu, Pedro A. Sánchez, Sofia Kantorovich, Yonggang Ke, Oleg Gang

**Affiliations:** † Department of Chemical Engineering, Columbia University, New York, New York 10027, United States; ‡ Computational and Soft Matter Physics, Faculty of Physics, University of Vienna, Boltzmanngasse 5, 1090 Vienna, Austria; § Department of Mathematical and Theoretical Physics, Institute of Mathematics and Natural Sciences, Ural Federal University, Ekaterinburg, 620026, Russia; ∥ MMM Mathematics-Magnetism-Materials, Research Platform, University of Vienna, Boltzmanngasse 5, 1090 Vienna, Austria; ⊥ Department of Applied Physics and Applied Mathematics, Columbia University, New York, New York 10027, United States; # Wallace H. Coulter Department of Biomedical Engineering, Georgia Institute of Technology and Emory University, Atlanta, Georgia 30322, United States; ∇ Center for Functional Nanomaterials, Brookhaven National Laboratory, Upton, New York 11973, United States

**Keywords:** Patchy particles, DNA nanotechnology, self-assembly, polymerization, phase behavior

## Abstract

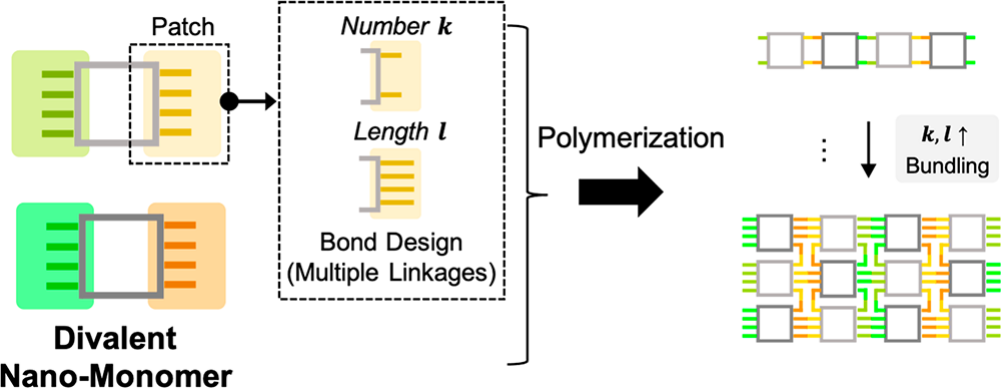

Assembly of nanoscale objects into linear architectures resembling molecular polymers is a basic organization resulting from divalent interactions. Such linear architectures occur for particles with two binding patches on opposite sides, known as Janus particles. However, unlike molecular systems where valence bonds can be envisioned as pointlike interactions nanoscale patches are often realized through multiple molecular linkages. The relationship between the characteristics of these linkages, the resulting interpatch connectivity, and assembly morphology is not well-explored. Here, we investigate assembly behavior of model divalent nanomonomers, DNA nanocuboid with tailorable multilinking bonds. Our study reveals that the characteristics of individual molecular linkages and their collective properties have a profound effect on nanomonomer reactivity and resulting morphologies. Beyond linear nanopolymers, a common signature of divalent nanomonomers, we observe an effective valence increase as linkages lengthened, leading to the nanopolymer bundling. The experimental findings are rationalized by molecular dynamics simulations.

Assembly of nano-objects into linear architectures offers an attractive route for forming “nanopolymers”,^1−9^ analogous to molecular polymers. These linear architectures are composed of inorganic nanoparticles^1,10,11^ or colloids,^12−14^ allowing for manipulation of plasmonic,^15−20^ magnetic,^21−24^ electronic^25−27^ and mechanical^28,29^ properties. One of the conceptually simple ways to create a nanopolymer is to use divalent particles, the so-called Janus particles, where two bonds, or binding patches are located on a particle’s opposite sides.^30,31^ This is one of the simplest variants of a general class of particles with anisotropic bonds, known as patchy particles.^32−34^ Janus particles have attracted much attention as versatile and designable building blocks at small scales, since they provide effective ways to control material fabrication into different morphologies.^14,30,35^

One of the key parameters dictating a morphology assembled from Janus particles is the relative size of each patch.^14,36,37^ For example, polymer-like, micelle-like, and layered structures were observed upon assembling divalent particles with two patches of similar or different areas, respectively.^36,38,39^ Since small molecules (1–2 nm) are often used as ligands to form patches, the specific details of their molecular architecture are typically important only to the degree that provides the interactions, while the molecular conformation states are typically neglected. The magnitude of these interactions approximately scales with patch area. However, if longer molecular moieties are grafted onto particles and grouped to create a nanoscale patch, new effects can arise in such cases due to a “dynamic” patch size, as provided by long-reaching molecular linkages. Most notably, if there is a physical mechanism for attraction (e.g., charge, hydrogen bonds, DNA hybridization, and so forth) among these molecular moieties located at different patches, the linkages might adopt conformations with a lower entropy since it can lead to the overall decrease of a free energy by maximizing cohesive energy. This allows for a far more complex interpatch connectivity than for a typical case of short ligands.

Such an effect was responsible, for example, for the observation of a spontaneous break of radial symmetry of interactions between nanorods grafted with long DNA strands^20^ due to low entropy penalty for stretching DNA linkages and for the formation of self-limited nanoparticle clusters^5^ due to the interactions of charged flexible polymers on their surfaces. The effect of linking motifs on 3D assembly have been extensively investigated for nano- and micron-sized particles^40,41^ but was not explored much for Janus particles. Thus, it is important to understand how interparticle interactions, mediated by long linkages, contribute to the morphologies formed by Janus particles, what is the underlying mechanism for particle assembly in such a scenario, and what is the relationship between patch structure (length and number of linkages) and resulting particle organization. Despite tremendous progress in constructing nanopolymers^3,42−45^ from functional nanoparticles, this aforementioned relationship is not well explored but it is important for predictable engineering of targeted nanoarchitectures.

Here, we investigate this general question by using a nanoscale system, where the particle’s size, shape, and design of patch structure can be fully controlled. DNA nanotechnology offers a methodology for tailorable fabrication of designed architectures,^41,46−57^ which is applied here to understand the physical mechanism of Janus particle’s interactions via multilinking bonds in different regimes of patch designs. A simplest structural morphology, one-dimensional (1D) chain array, can be realized using DNA nanomonomers (e.g., tiles, origami) with patches on opposite sides of a nanomonomer,^19,27,58,59^ resembling conventional polymerization. This regime has been only mapped out for polymerizing divalent patchy particles via short linkages.^52,53^ In our study, the divalent nanomonomers are DNA-based cuboids, so-called DNA nanochamber (DNC),^52^ on which length-adjustable and number-prescribed single-stranded DNA (ssDNA), capable of forming molecular linkages, are zoned as two patches (Figure 1). Our present work focuses on uncovering the relationship between the patch structure (namely, the number and length of single-stranded (ss) DNA in one patch, and their placement within the patch), resulting assembly kinetics and morphologies.

**Figure 1:**
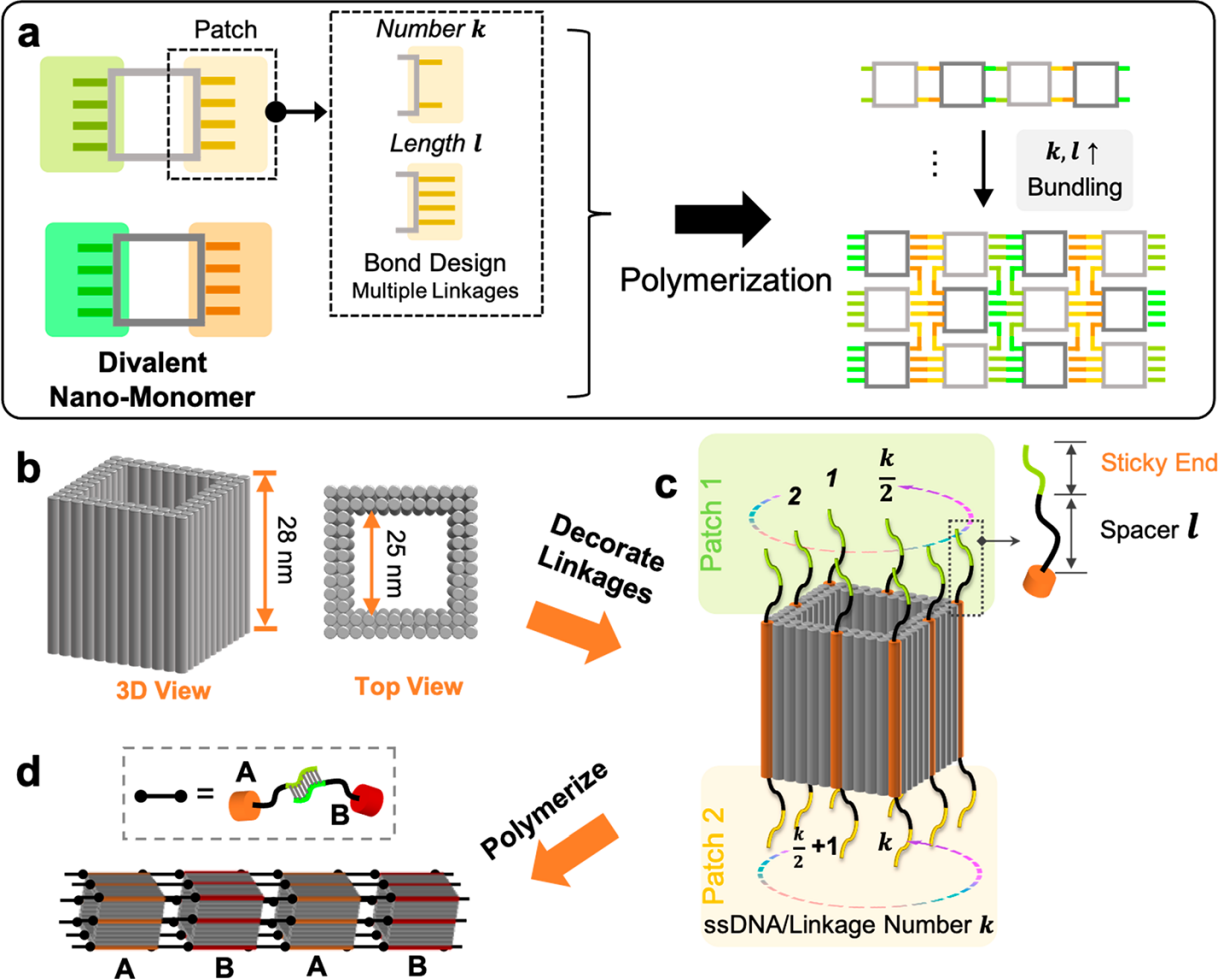
Schematic of assemblies from divalent cuboid nanomonomers with patches composed of DNA linkages on opposite faces. (a) Schematics of a design of divalent nanomonomer with multiple molecular linkages, where the number and length of linkage can be adjusted. The nanomonomers can form single and bundling nanopolymers. (b) Illustration of a cuboid DNA nanomonomer. (c) The top and bottom faces of a cuboid nanomonomer are decorated with multiple ssDNA containing distinct overhangs (sticky ends). The ssDNA can hybridize with their complementary strands from other patches and constitute longitudinal double-stranded linkages. Nanomonomer reactivity can be tuned through altering ssDNA number *k* and length *l*, as shown. (d) The growth of a nanopolymer by connecting two complementary patches of nanomonomers, A and B.

Our study reveals that for divalent nanomonomers: (i) polymerization kinetics initially follows the classic step-growth polymerization theory but later deviates due to the stagnant diffusion of preformed “nano-oligomer”; (ii) polymerization is also subject to nanomonomer reactivity, which is determined by both ssDNA number (*k*) and length (*l*); (iii) the formation of well-aligned “bundle” architectures with an ordered organization is observed at larger *k* and *l*, where the effective valence increases above its nominal divalent value due to multiconnectivity provided by longer linkages.

Given the rich assembly behavior of cuboid blocks,^15,50,52,60−67^ we explore here the morphology of patched cuboids, a cuboid nanomonomer (DNC)^52^ to probe nanopolymer polymerization. DNC is created by DNA origami technology (Figure 1b), where an opening cavity (25 nm × 25 nm × 28 nm) is enclosed with double-layer DNA helix walls. The sequence-specific ssDNA are placed at two opposite faces of the nano-monomer (top and bottom, Figure 1c) and can be grouped as patches to provide directional intermonomer bonds. To constitute molecular linkage in one patch, a part of ssDNA, composed of a spacer and an overhang, the so-called “sticky end” capable of hybridization with its complement, is extended from one of the two terminations of the selected DNA helix. To prevent undesired random binding between ssDNA and to maintain an orientation of cuboids, all sticky ends bear distinct eight-base sequences to form “polychromatic” linkages.^52,53^ In our design, the possibility of binding between complementary nanomonomers, referred to as nanomonomer reactivity, is determined by two parameters: (i) the number of ssDNA per nanomonomer (*k*) that are symmetrically distributed on the two decorated faces; (ii) the number of thymine nucleobases (poly-T) in a spacer (*l*) of the ssDNA, which provides flexibility to the formed linkages. We refer to a specific patch design of a nanomonomer with ssDNA number *k* and length *l* as *M*_*k*_^*l*^.

To induce nanopolymer assembly, we mixed an equal molar ratio of nanomonomers with complementary sticky ends (nanomonomers of types A and B, Figure 1d). The kinetics of nanomonomer assembly resembles step-growth polymerization of molecular monomers. To explore such a polymerization on the nanoscale, we carried out the polymerization of *M*_64_^20^ (*l* = 20 and *k* = 64) at a constant temperature (40 °C). In this patch design, each corner and middle edge of a nanomonomer is decorated with four binding strands bearing a spacer of 20 poly-T (inset, Figure 2a(i)). Using images obtained by negatively stained transmission electron microscopy (TEM), we monitored the growth of nanopolymers (Figure 2a) and statistically quantified the fraction of observed polymer species with a degree of polymerization *X*_*n*_, defined by the number of nanomonomers in the nanopolymer (Figure 2b). Note that the kinetic study is performed at a constant temperature to exclude the influence of temperature variables, while the structures discussed in Figures 3 and 4 are formed under an adjusted annealing processes. Upon mixing (within 2 min), the majority of nanomonomers (69%) remain unbonded with only a small portion of dimers (23%) and trimers (8%). An increase in *X*_*n*_ over time is then observed, yielding nano-oligomers containing 3–5 nanomonomers (61%) in 1 h and nanopolymers with ≥5 nanomonomers (85%) in 24 h (Figure 2b).

**Figure 2:**
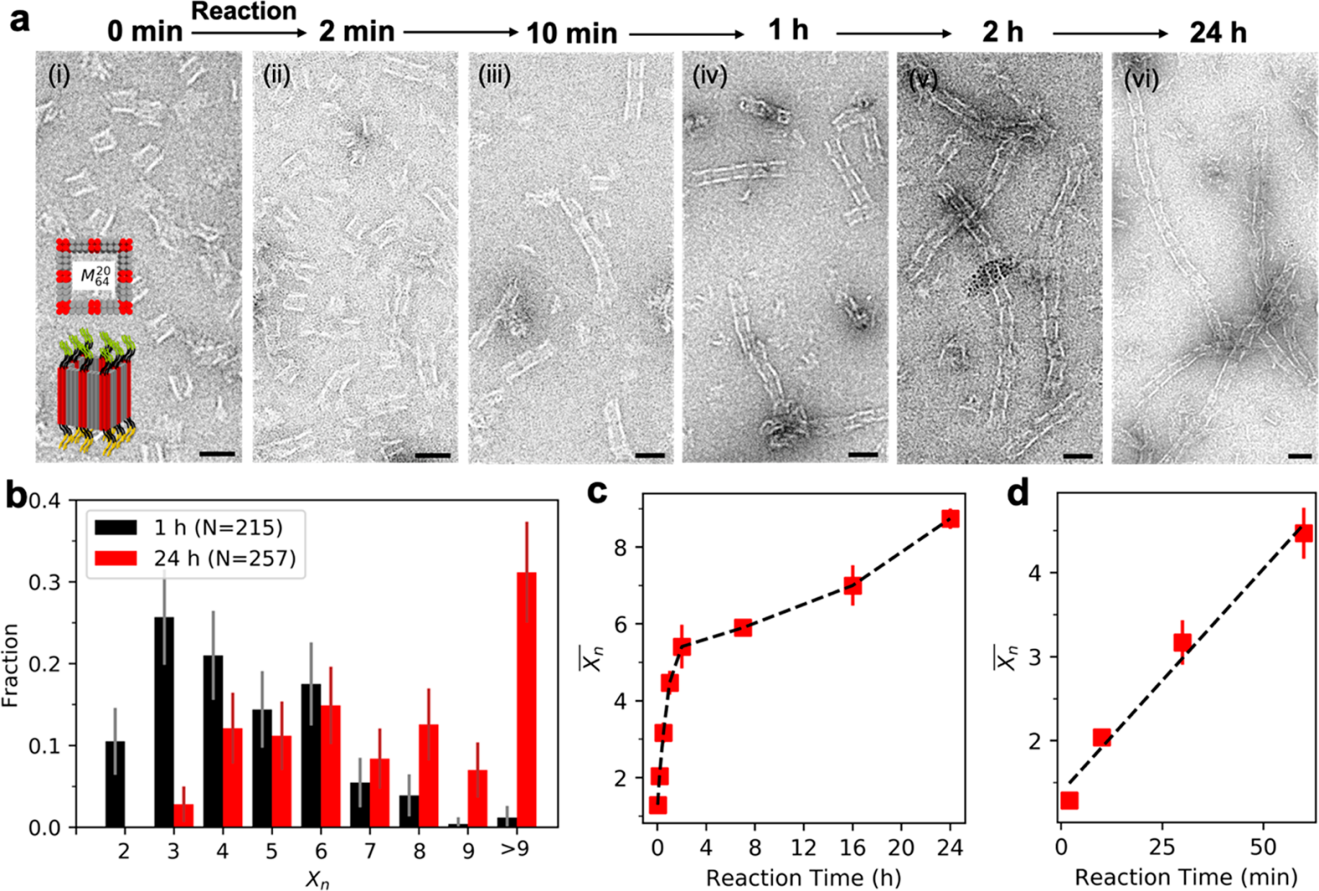
Polymerization kinetics of nanomonomers. (a) TEM images showing the time-dependent growth of nanopolymers for the *M*_64_^20^ design (inset). (i) Zero minutes before the polymerization reaction; (ii) 2 min; (iii) 10 min; (iv) 1 h; (v) 2 h; (vi) 24 h. Scale bars: 50 nm. (b) Fractions of nanopolymers with the degree of polymerization (*X*_*n*_) for the *M*_64_^20^ design at 1 h (*N* = 1799) and 24 h (*N* = 1147). Error bars represent 95% confidence interval. (c,d) Dependency of number-average *X*_*n*_ (*X̅_n_*) on reaction time in (c) 24 h and (d) 1 h. Dashed line in (d) presents linear regression. Error bars denote s.d. of triplicates.

**Figure 3:**
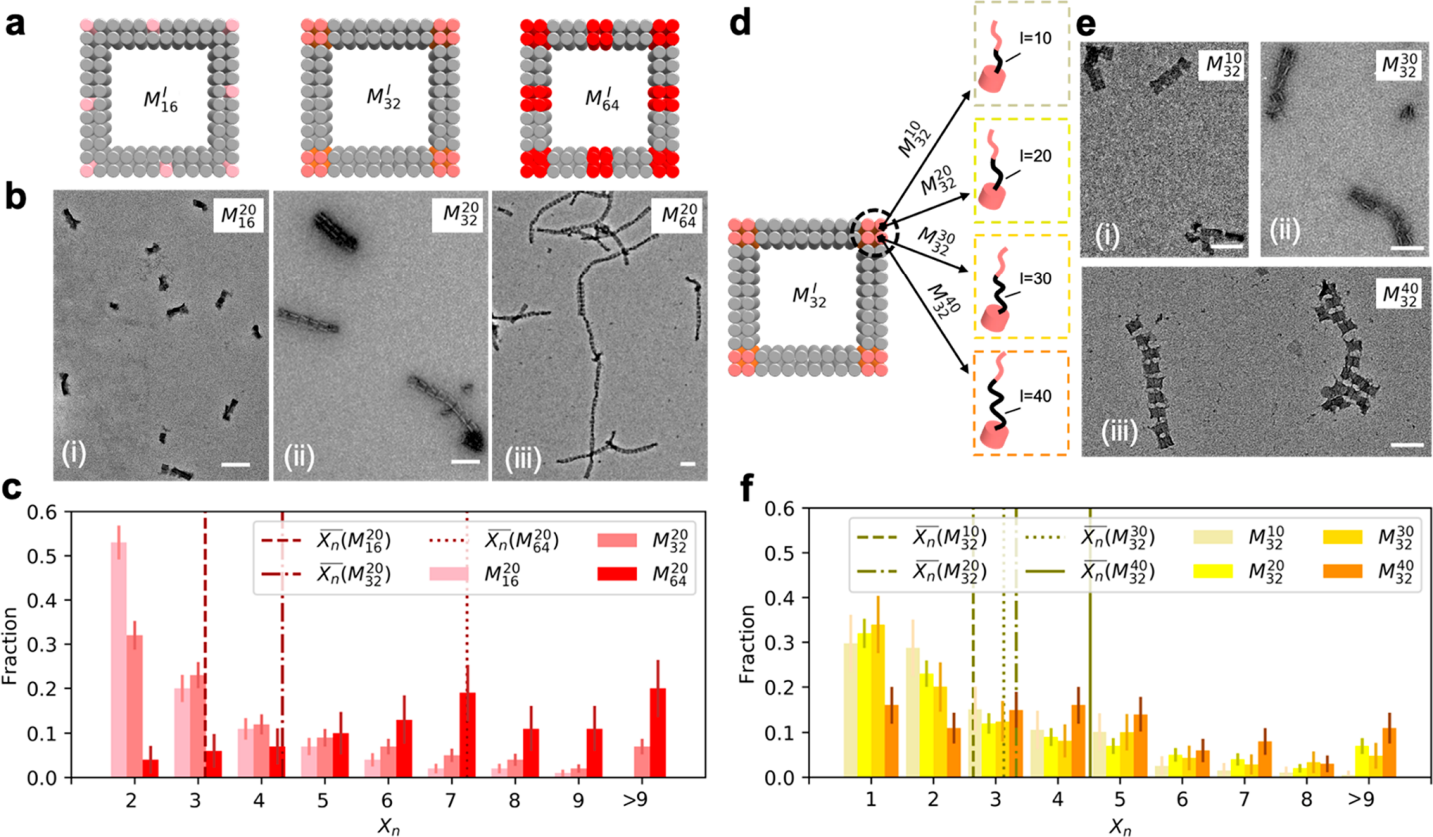
Effect of nanomonomer reactivity on polymerization. (a) Top-view illustration of *M*_16_^*l*^, *M*_32_^*l*^, and *M*_64_^*l*^ designs; red-colored helices are decorated with ssDNA. (b) TEM images of resultant nanopolymers synthesized from (i) *M*_16_^20^, (ii) *M*_32_^20^, and (iii) *M*_64_^20^. (c) Fractions of nanopolymers with *X*_*n*_ using nanomonomer designs *M*_16_^20^ (*N* = 647), *M*_32_^20^ (*N* = 782), and *M*_64_^20^ (*N* = 148). Dashed lines denote *X̅_n_*(*M*_16_^20^) = 3.11, *X̅_n_*(*M*_32_^20^) = 4.33, *X̅_n_*(*M*_64_^20^) = 7.24. (d) Zoomed-in, top-view illustration of the patch designs for *M*_32_^*l*^. (e) TEM images of nanopolymers synthesized from (i) *M*_32_^10^, (ii) *M*_32_^30^, and (iii) *M*_32_^40^. (f) Fractions of nanopolymers with *X*_*n*_ using *M*_32_^10^ (*N* = 198), *M*_32_^20^ (*N* = 782), *M*_32_^30^ (*N* = 209), and *M*_32_^40^ (*N* = 317) designs. Dashed lines denote *X̅_n_*(*M*_32_^10^) = 3.64, *X̅_n_*(*M*_32_^30^) = 4.13, *X̅_n_*(*M*_32_^40^) = 5.52. Note that to make an adequate comparison, bundling structures are excluded from statistical analysis in (c,f). The reaction conditions contain cooling A and B nanomonomers from 50 to 20 °C at a rate of 0.3 °C/h. Scale bars: 100 nm. All error bars represent 95% confidence intervals.

**Figure 4:**
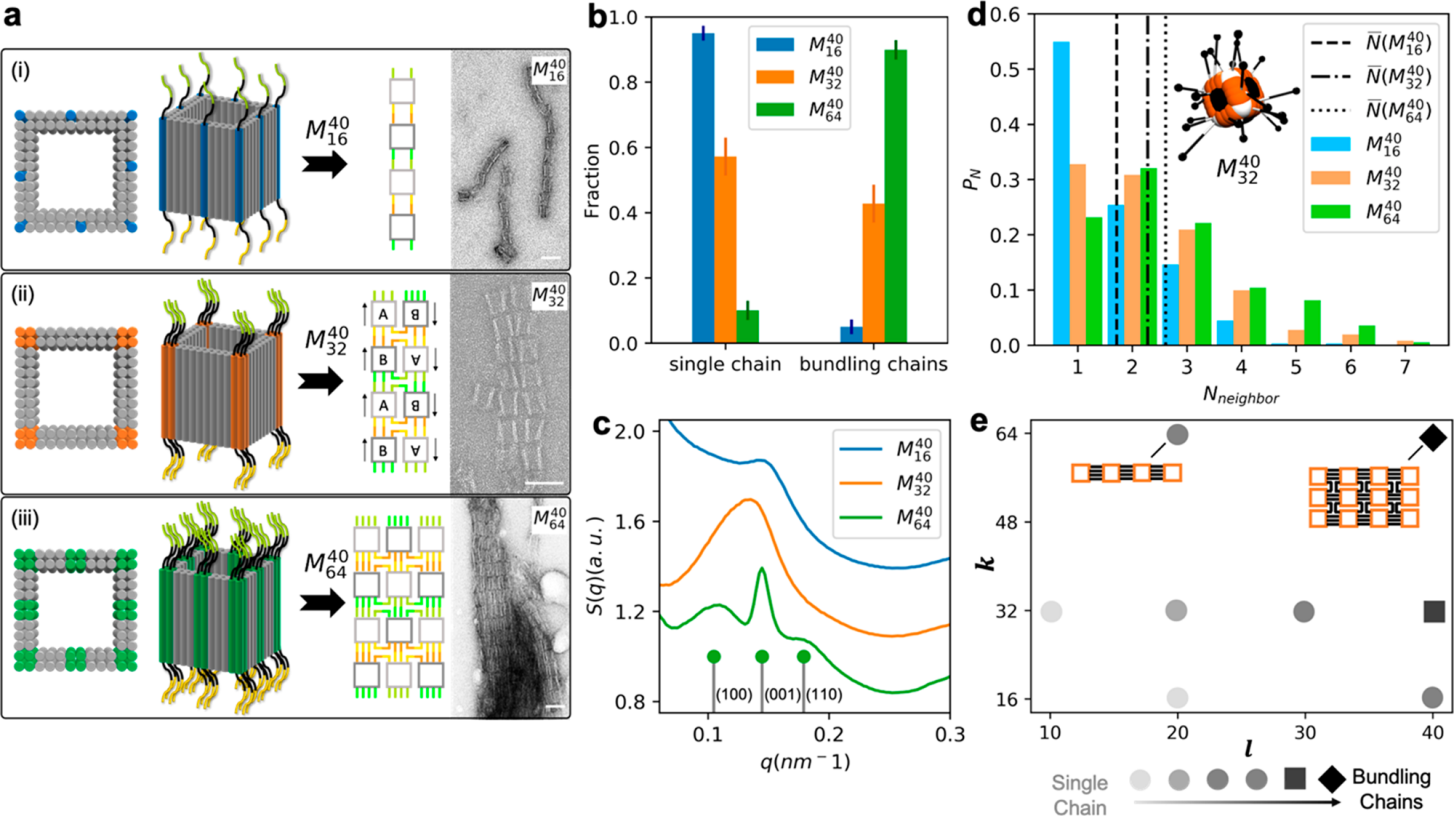
Formation of bundled nanopolymers. (a) Patch design and corresponding TEM images of resultant nanopolymers by (i) *M*_16_^40^, (ii) *M*_32_^40^, and (iii) *M*_64_^40^. Scale bars: 50 nm. (b) Fractions of single nanopolymer chains and bundling chains present in the three systems where only nanopolymers with *X*_*n*_ ≥ 5 are counted: *M*_16_^40^ (*N* = 358), *M*_32_^40^ (*N* = 278), and *M*_64_^40^ (*N* = 398). Error bars represent 95% confidence interval. (c) Structure factors of the three systems obtained by SAXS. The peak indexing at the bottom denotes the first three peak positions of an ideal 3D primitive tetragonal lattice (*a* = *b* = 58.8 nm, *c* = 43.5 nm). (d) Probability (*P*_*N*_) of finding a monomer bound with neighboring particles *N*_neighbor_, mimicking *M*_16_^40^, *M*_32_^40^, and *M*_64_^40^ after simulated annealing. Inset: schematic representation of DNC (*M*_32_^40^). Dashed lines: average value of neighbors, *N̅*(*M*_16_^40^) = 1.71, *N̅*(*M*_32_^40^) = 2.28, and *N̅*(*M*_64_^40^) = 2.61. (e) Morphological phase diagram of nanopolymers in *k* and *l*. Darker shade of gray color corresponds to longer nanopolymer; circle to square to diamond denotes the phase transition from single chain to coexistent morphology to bundled chains.

We further analyze the evolution of nanopolymer growth by plotting the dependence of the number-average degree of polymerization (*X̅_n_*) on reaction time (*t*) (Figure 2c,d). The reaction process can be divided into two stages. In the first stage (*t* ≤ 1 h), *X̅_n_* increases proportionally with *t*, which is a characteristic of a reaction-controlled step-growth polymerization. This scenario is consistent with the classic Flory model, where *X̅_n_* ∼ [*M*]_0_*kt*, [*M*]_0_ is the initial concentration of nanomonomers (in our case, [*M*]_0_ = 16 nM). In the second stage (*t* > 1 h), the polymerization reaction becomes moderate, corresponding to a nonlinear relationship. It indicates that in this regime the assumption of constant reactivity of functional groups throughout the polymerization, as in the Flory model, is not fully realized. We hypothesize that (i) as a nanopolymer grows, the diffusion of nano-oligomers becomes stagnant; (ii) by design, polychromatic linkages enforce strict intermonomer orientations during polymerization. Therefore, in the second stage translational diffusion of nano-oligomers and rotational diffusion of nanomonomers retard polymerization rate.

In addition to kinetic factors, nanomonomer reactivity also affects the growth of nanopolymers. In order to investigate the effect of reactivity on polymerization, we systematically change the *k* (Figure 3a) and *l* (Figure 3d). First, the influence of *k* is examined using three different designs in which ssDNA are encoded onto block faces with a 4-fold rotational symmetry: in *M*_16_^*l*^, eight of ssDNA are separately positioned at corners of opposite faces (one linkage per corner), while the remaining eight ssDNA are positioned in the middle of each edge (one linkage per edge); in *M*_32_^*l*^, 32 ssDNA are equally distributed at eight corners (four linkages per corner); in *M*_64_^*l*^, 64 ssDNA are located as described above in Figure 2. Figure 3b presents TEM-observed morphologies of nanopolymers constructed from nanomonomers with *l* = 20 and different *k*, under consistent reaction conditions. Note that whereas the *M*_*k*_^20^ systems were previously reported,^52^ we further quantitatively analyze these systems by TEM in this work (Supporting Information Part 1b). The representative results (Figure 3b) indicate that the length of resulting nanopolymers increases with increasing *k*. Such a trend is supported by the *X*_*n*_ distribution for the three corresponding designs (Figure 3c). For example, in the case of *X*_*n*_ ≥ 5 number fraction of the nanopolymers enhances from 35% to 56% and to 96% as *k* rises from 16 to 32 and to 64, respectively. *X̅_n_* further verifies the positive correlation between the length of nanopolymers and the density of linkages: *X̅_n_*(*M*_16_^20^) = 3.11 < *X̅_n_*(*M*_32_^20^) = 4.33 < *X̅_n_*(*M*_64_^20^) = 7.24. Such correlation is ascribed to the alteration of overall hybridization energy proportional to *k*, resulting in longer nanopolymer in *M*_64_^20^ than *M*_16_^20^. Furthermore, the increased number of ssDNA also contributes to a higher binding probability of nanomonomers with larger *k* by partially connecting to linkages.

Next, we investigate the effect of *l* on the nanomonomer reactivity, by fixing *k* but varying *l* in a range of 10 to 40 (Figure 3d). We choose *M*_32_^*l*^ designs as a representative system to probe the correlation between *l* and *X*_*n*_ based on TEM imaging (Figure 3e). Statistical results of *X*_*n*_ distributions for these systems exhibit the track of *X̅_n_*(*M*_32_^10^) < *X̅_n_*(*M*_32_^20^) ≈ *X̅_n_*(*M*_32_^30^) < *X̅_n_*(*M*_32_^40^) (Figure 3f). This trend should be attributed to two entropic factors: (i) conformational entropy of longer ssDNA is higher than these of shorter ones; (ii) the effective cross-section for intermonomer binding is expanded with increased *l*. Together, both effects facilitate the nanomonomer reactivity, yielding a higher probability of polymerization with larger *l*.

It is worth noting that beyond the longitudinal growth of polymers, we occasionally observed lateral growth of nanopolymers with a different morphology, as shown by *M*_32_^40^ design (Figure 3e(iii)), which is barely present in other systems with shorter *l*. Therefore, we first hypothesize that a long linkage may facilitate the generation of branched nanopolymers, analogous to molecular branched polymers where side chains can randomly grow out from the main chain. To verify this hypothesis, we design three different types of nanomonomers with long linkages (*l* = 40), varying *k* from 16 to 64. Figure 4a displays these nanomonomers and corresponding morphologies obtained by TEM. Surprisingly, instead of forming branched architectures, we observe the formation of either long nanopolymers or bundles of ordered chains. Specifically, we notice a series of morphological transitions from single chains (*M*_16_^40^) to double chains (*M*_32_^40^) by antiparallelly aligning nanopolymers based on the directional bonds (Figure 4a(ii)) and to multichain bundles (*M*_64_^40^). We further quantify our observation by analyzing a large number of nanopolymers examined by TEM (Figure 4b and Figures S4–6): in *M*_16_^40^, single chains (95%) are in majority; in *M*_32_^40^, single chains (57.2%) and bundles (42.8%) are evenly contested; in *M*_64_^40^, bundles (89.9%) dominate.

To further investigate the organization of nanomonomers, we applied *in situ* small-angle X-ray scattering (SAXS). Figure 4c summarizes the obtained structure factors (*S*(**q**), **q** is a wavevector) for *M*_16_^40^, *M*_32_^40^ and *M*_64_^40^ designs. *S*(**q**) profile of *M*_16_^40^ exhibits a single peak, suggesting a 1D array with an average intermonomer distance of 43.5 nm. Strikingly, *S*(**q**) of *M*_64_^40^ shows three peaks with positions corresponding to (100), (001), and (110) crystalline planes of a primitive tetragonal lattice (*a* = *b* = 58.8 nm, *c* = 43.5 nm) with resolution-corrected correlations lengths of 172 nm for the (100) direction and 722 nm for the (001) direction, unambiguously identifying lateral packing of nanopolymers. We thus emphasize that the assembly of bundled nanopolymers actually presents an elongated morphology with 3D lattice of DNA cuboids rather other disordered branching nanopolymers. As an intermediate design between *M*_16_^40^ and *M*_64_^40^, *M*_32_^40^ exhibits a merged broad peak, representing a transitional stage between single and bundling chains.

We propose that the unique binding characteristics of nanomonomers are determined by multilinking bonds, which is different from its molecular counterparts, and it should be the main factor responsible for the formation of bundles with 3D lattice structure. In order to elucidate the mechanism underlying the featured bonds, we employ molecular dynamics simulations using a coarse-grained bead–spring approach.^65^ In our simulations, the DNC is represented by cubic unit to which elastic band-like symmetric “ssDNA” with sticky ends are attached (Figure 4d, inset, Supporting Information Part 3). We analyze the cluster structure formed by the simulated monomers with different *k*. The topology of the monomer clusters is characterized by the coordination number *N*_neighbor_, namely the number of close neighbors to which each monomer binds, which also indicates an effective valence value of target monomers (Figure 4d). Note that for linear structures, *N*_neighbor_ is between 1 and 2; *N*_neighbor_ above 2 suggests bundling or branching. We then compare *N̅* (number-average of *N*_neighbor_) and observed morphologies with the characteristics of single chain formation for low *k* and bundling of chains for larger *k*: *M*_16_^40^ with *N̅* = 1.71 performs a linear polymerization confirmed by Figure 4b; *M*_32_^40^ with *N̅* = 2.28 hints the occurrence of partially branching or bundling; *M*_64_^40^ with *N̅* = 2.61 strongly suggests a more significant cross-linking behavior among nanomonomers over *M*_32_^40^. Such essential agreement between experimental observation and simulation explains the prerequisite role of multilinking bonds on monomer valence in three dimensions.

In addition to multiple linkages and the resulting clusters, the effect of cubic monomer shape should not be disregarded, and it may captain the formation of well-ordered bundles from the clusters. Indeed, in the self-assembly process, the interplay of a block’s shape and entropic effect yields complex assembling pathways, especially for blocks with anisotropic shapes.^58,68^ Therefore, the packing of cuboid nanomonomers in bundling nanopolymers may minimize the steric hindrance from neighbors and maximize the global entropy. Thus, based on the simulation results we further speculate that the two structural features of nanomonomers contribute to the unusual morphological transition in the following ways: (i) the longer and denser linkages result in an increased effective nanomonomer’s valence; (ii) the cuboid-shape feature orients the clusters in a way of well-ordered bundles due to the steric hindrance from nanomonomer.

To parametrize the observed polymeric structures, a morphological phase diagram summarizing all investigated patch designs (*l* and *k*) is constructed (Figure 4e). With low *l* and *k*, nanomonomer connection is restricted, resulting in oligomer-dominant structures. Increasing both *l* and *k* significantly enhances the efficiency of polymerization due to boosted nanomonomer reactivity, giving rise to long nanopolymers. A morphological transition from discrete chains to bundled chains occurs at *l* = 40, where bundles dominate over single chains in nanopolymer populations. Remarkably, in the case of *l* = 40 and *k* = 64, we observe relatively well-ordered bundled chains whose *S*(**q**) is comparable to the one expected for a primitive 3D tetragonal arrangement. On the basis of the aforementioned mechanism of bundling, the flexible and multiple linkages must exist before the formation of bundling, which explains this morphological transition taking place in the regime of large *l* and *k*.

The formed 3D bundles of nanopolymers with internal organization are dramatically different from molecular polymers with randomly branched side chains. This difference can be primarily attributed to (i) limited malleability of nanopolymers and (ii) the established multilinking bonds between nanomonomers. “Polychromatic” nature of DNA-encoded bonds enforces either longitudinal “face-to-face” or lateral “side-to-side” orientations, which allows to maximize hybridization of ssDNA. Furthermore, in contrast to the conventional polymers where divalent monomers interact typically through pointlike contact nanomonomers can engage many molecular moieties located at the patches. This significant difference diversifies assembly behavior, leading to new regimes where effective valence will increase above two. It results in nanopolymer “bundling”, which has no direct analogy with the molecular scale systems. We stress that two types of “bonds” are occurring in the presented nanoscale system: (i) one type is a molecular bond formed by two complementary ssDNA, termed as linkage in our study, and (ii) intermonomer patch-to-patch bond resulting from grouping of the first molecular bonds. While the “second bond” is mainly responsible for nanopolymer formation, the longer “first bond” affords connection among more than two patches, reflected as the valence increase above two.

In summary, we investigate in detail the polymerization behavior of a divalent nanomonomer with two opposite patches, namely cubic DNA nanochamber with longitudinal multilinking bonds created by hybridization of ssDNA. The polymerization kinetics at the initial stage resembles molecular analogs while the later stage is governed by a diffusion-controlled processes. We show that the nanomonomer reactivity is primarily controlled by two patch parameters, ssDNA number *k* and length *l* on a nanomonomer, whose effects on the polymerization behavior are uncovered; increasing either *l* or *k* results in the formation of longer nanopolymers. Remarkably, we observe a morphological transition from single discrete nanopolymer chains to bundles of ordered chains at larger *l* and *k*. A coarse-grained bead spring model is then established to decipher this unusual behavior. The experimental and theoretical agreement reveals that with larger *l* and *k* the lateral connection between nanomonomers is driven by the interplay between patch design and cuboid shape of a monomer, resulting in the elongated morphology of 3D lattice. This work bridges a gap between molecular polymers and nanopolymers toward a comprehensive understanding of the factors controlling assembled state and for constructing complex ordered arrays of nanoobjects through linear assembly motifs.

## Supporting Information Available

Additional experimental details, materials, and methods, including synthesis of DNC and nanopolymer; details of SAXS measurements; TEM images; DNA sequences; Molecular Dynamics simulation of DNC (PDF)
