# Supplementary material for: Divalent Multilinking Bonds Control Growth and Morphology of Nanopolymers
Source: Nano Lett. 2021 Oct 14;21(24):10547–54. doi: 10.1021/acs.nanolett.1c03009 (PMC8704199; doi:10.1021/acs.nanolett.1c03009)
Supplement: Supplementary file 1 — nl1c03009_si_001.pdf [file nl1c03009_si_001.pdf]

Divalent Multi-Linking Bonds Control Growth and Morphology of Nano-Polymers

Yan Xiong[1‡], Zhiwei Lin[1‡], Deniz Mostarac[2,4], Brian Minevich[1], Qiuyuan Peng[5], Guolong Zhu[1], Pedro A. Sánchez[2], Sofia Kantorovich[2,3,4], Yonggang Ke[6], and Oleg Gang[1,5,7*]

[1]Department of Chemical Engineering, Columbia University, New York, NY 10027, USA

[2]Computational and Soft Matter Physics, Faculty of Physics, University of Vienna, Vienna, Austria

[3]Department of Mathematical and Theoretical Physics, Institute of Mathematics and Natural Sciences, Ural Federal University, Ekaterinburg, Russia

[4]MMM Mathematics-Magnetism-Materials, Research Platform, University of Vienna, Vienna, Austria

[5]Department of Applied Physics and Applied Mathematics, Columbia University, New York, NY 10027, USA

[6]Wallace H. Coulter Department of Biomedical Engineering, Georgia Institute of Technology and Emory University, Atlanta, Georgia 30322, USA

[7]Center for Functional Nanomaterials, Brookhaven National Laboratory, Upton, NY 11973, USA

[‡] These authors contributed equally to this work.

Table of Contents

Part 1. Materials and Methods **2**

a. Synthesis of DNA Nano-Monomer **2**

b. Assembly of DNA Nano-Monomers **2**

c. Preparation of DNA-Grafted Nanoparticle **2**

d. Small Angle X-Ray Scattering (SAXS) **3**

e. Transmission Electron Microscopy (TEM) **4**

Part 2. DNA Sequences **13**

a. DNA Nano-Chamber **13**

b. Sequences for DNA Binding Strands **17**

Part 3. Simulation of Nano-Monomer Using Molecular Dynamics **26**

a. Raspberry Model and Steric Interactions **26**

b. Simulating Self-Assembly of Monomers **28**

Part 1. Materials and Methods

a. Synthesis of DNA Nano-Monomer

DNA nano-chamber (DNC), folded with scaffold (M13mp18, purchased from Bayou Biolabs) and 224 oligonucleotide staples, is employed as the DNA nano-monomer to polymerize nano-polymers. In order to synthesize the DNCs, 10 nM scaffold was mixed with 50 nM staples in a buffer containing 1 mM EDTA, 5 mM Tris and 16 mM MgCl2. This mixture was annealed by rapid heating up to 80 °C and keeping for 5 min, then cooling to 65 °C at the rate of 1 °C/min, afterward, slowly cooling to room temperature at the rate of 1 °C/h.

After annealing process, the sample was subjected to column-based purification (Amicon Ultra-Centrifugal Filter, 100K). The column filter was wetted by 0.5X TBE with 10 mM $MgCl_2$ buffer and spined at 2k rcf for 10 min. Next, the post-annealing sample was put into the wet column and spined at 2k rcf for 10 min, followed by removal of the bottom fraction. Then fresh buffer with the equal volume of removed bottom fraction was added to the column with spinning process at 2k rcf for 10 min. After five repeats of the buffer-exchanging procedure, the purified DNC at the top fraction of the column was collected. Subsequently, the concentration of DNC was measured by microvolume spectrophotometers (Thermo Scientific NanoDrop™ Lite Spectrophotometer).

b. Assembly of DNA Nano-Monomers

To polymerize nano-polymer from the purified DNCs, equal mole of two types DNCs A and B were mixed for further annealing. If the sample required framed AuNPs for SAXS measurement, DNA-grafted AuNP was includes into this mixture as well (see Part 1c-d). The prescribed annealing procedure contains cooling from 50 °C to 20 °C at a rate of 0.3 °C/h.

c. Preparation of DNA-Grafted Nanoparticle

The grafted DNA, thiolated oligonucleotide purchased from IDT, was first reduced by TCEP (tris[2-carboxyethyl] phosphine) with a ratio of 1:100 in distilled water. 10 nm gold nanoparticle (AuNP) purchased from Nanopartz was mixed with the reduced monothiol-modified DNA with a molar ratio of 1 AuNP to 300 oligonucleotides. The mixture was aged for 1.5 h and was mixed with surfactant solution (SDS, final concentration is 0.01%) to keep particle suspended. Next, the solution was buffered into 10 mM phosphate buffer, followed by slowly adding NaCl to achieve a final concentration of 0.3 M NaCl. The final solution was aged overnight at room temperature. Free oligonucleotides were removed by centrifuging the aged sample (15k rpm, 1h),

removing 90% supernatant, keeping the precipitation and compensating equal volume of phosphate buffer with 100 mM NaCl. After four repeats of washing process, we collected the precipitation of DNA-grafted AuNP. The concentration of DNA-grafted AuNP was then characterized using ultraviolet–visible spectrophotometry. The extinction coefficient of 10 nm AuNP at 520 nm is $3.64 \times 10^8\ M^{-1}cm^{-1}$.

In order to frame the DNA-grafted AuNP into the inner chamber of DNC, we mixed the AuNP with column-purified DNC which contains the interior sticky ends for anchoring AuNP (see Part 2) with a molar ratio of 2.5:1 and annealed the mixture following the procedure in Method section.

d. Small Angle X-Ray Scattering (SAXS)

The SAXS measurements were conducted at one of the user end-stations, or beamlines at the National Synchrotron Light Source II (NSLS-II) at Brookhaven National Laboratory, located in Upton, NY. More specifically, these measurements were done at the Complex Materials Scattering (CMS) beamline. Details related to these measurements can be found in the table below. To prepare the samples for SAXS, 15 µL nano-polymer sample, where gold nanoparticle (AuNP) was encaged into DNC to provide sufficient signal, was loaded into the bottom of quartz capillary and sealed by wax. After 2 h, black-reddish precipitation can be observed at the bottom of capillary where the beam spot should position for SAXS measurements.

Table 1. CMS Beamline Experimental Setup

Beamline	11-BM CMS
Photon Energy (keV)	13.5
Horizontal × Vertical Beam size (µm × µm)	200 x 200
Approximate Flux (photons/sec)	1011
Sample-to-Detector Distance (m)	5.05
Detector Manufacturer	Dectris
Detector Model	Pilatus 1M
Detector Pixel Size (µm x µm)	172 x 172

The 2D area images were integrated into one-dimensional (1D) I(q) scattering curves as a function of the scattering vector, q, whereby q=4 π/λ sin(θ/2) with λ and θ being the wavelength of the incident X-rays and the full scattering angle, respectively. These curves were generating by using an azimuthal integration library developed in Python, known as pyFAI and is available for

download on Github. The resultant 1D curves span from roughly 0.03 nm^{-1} to 1 nm^{-1} with a resolution of 0.002 nm^{-1}. The experimental S(q) was calculated by dividing the obtained I(q) 1D curves by the form factor or P(q) for the corresponding particles used in the sample preparation.

Correlation lengths in directions of specific reflecting planes are calculated by fitting the peaks in the S(q) data to a Lorentzian distribution. The instrumental resolution of the beamline is subtracted from the full-width at half-max (FWMX) of the fit peaks and then converted from $Å^{-1}$ to nm.

e. Transmission Electron Microscopy (TEM)

To prepare samples for TEM characterization, copper mesh grids (purchased from Ted Pella) were treated with glow discharge for 1 min. Next, nano-polymer samples were fully mixed and 5 μL sample was pipetted and loaded onto the treated grids for 2 min, followed by wicking away excess liquid through filter paper. Then 4 μL uranyl acetate was loaded onto the grid for 10 s to stain target DNA structures. This grid was loaded into single tilt holder of TEM (Titan Themis 200 kV TEM).

Note that although consistent sample preparations were carried out, negatively and positively-stained structures were simultaneously imaged by TEM. In the negatively-stained case, the DNCs were stained lighter than the background and mainly retained their 3D geometry. On the other hand, in the positively-stained case, the DNCs were stained darker than the background and distorted under such condition, resulting in a shape of flattened cube.

Representative images of the studied systems are shown in the following pages.

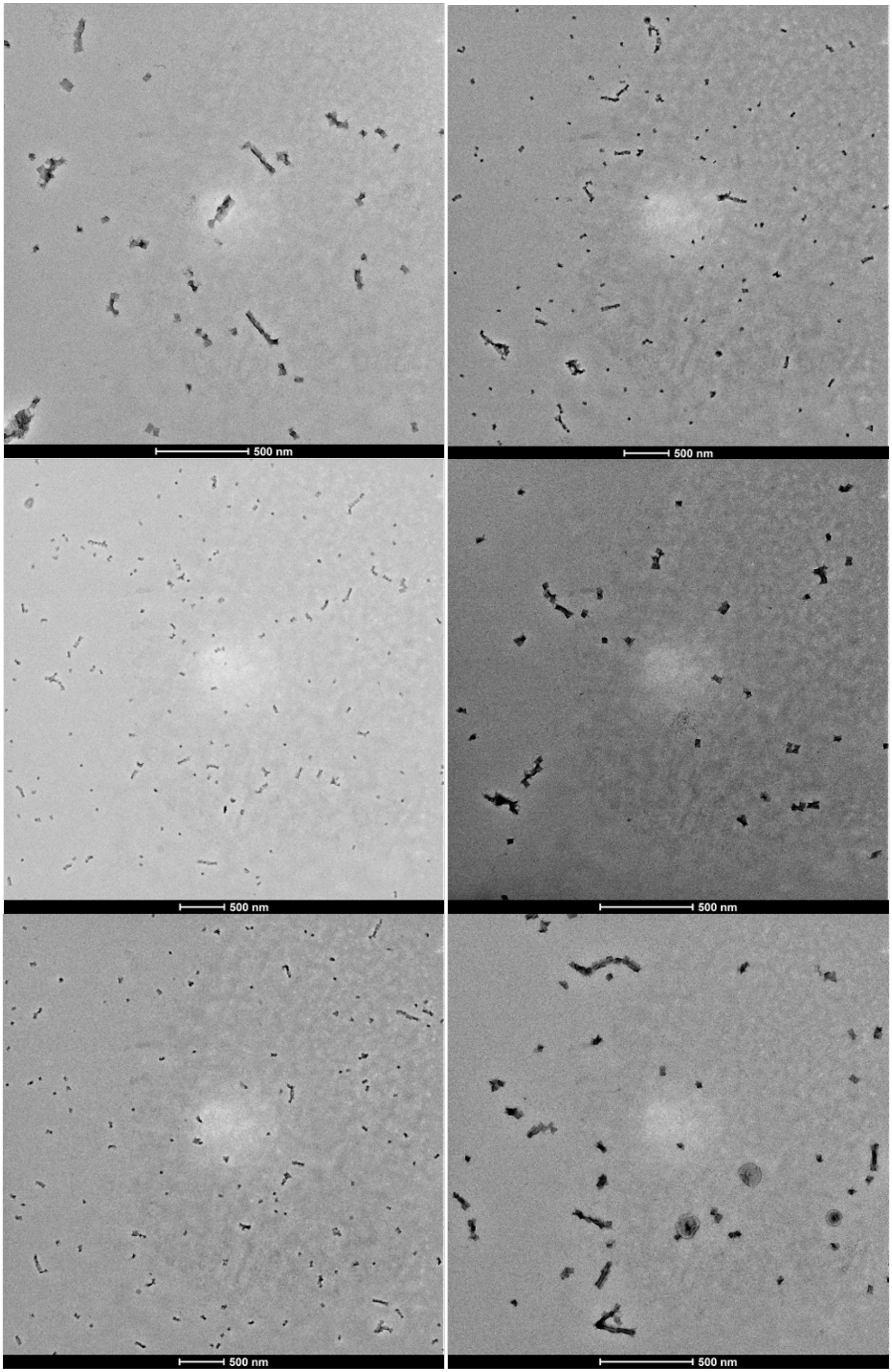

Figure S1. Representative TEM images of M_{16}^{20}.

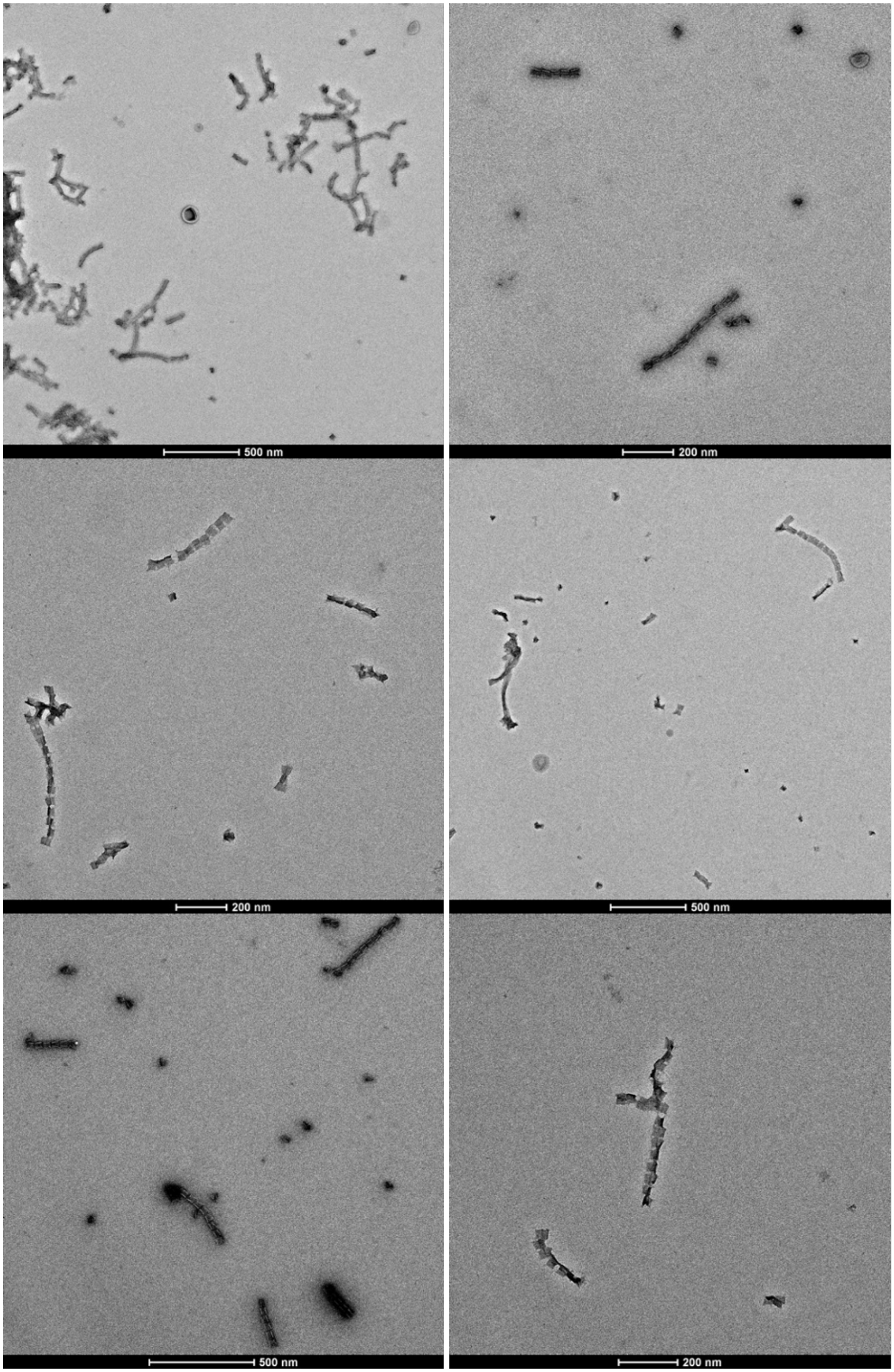

Figure S2. Representative TEM images of M_{32}^{20}.

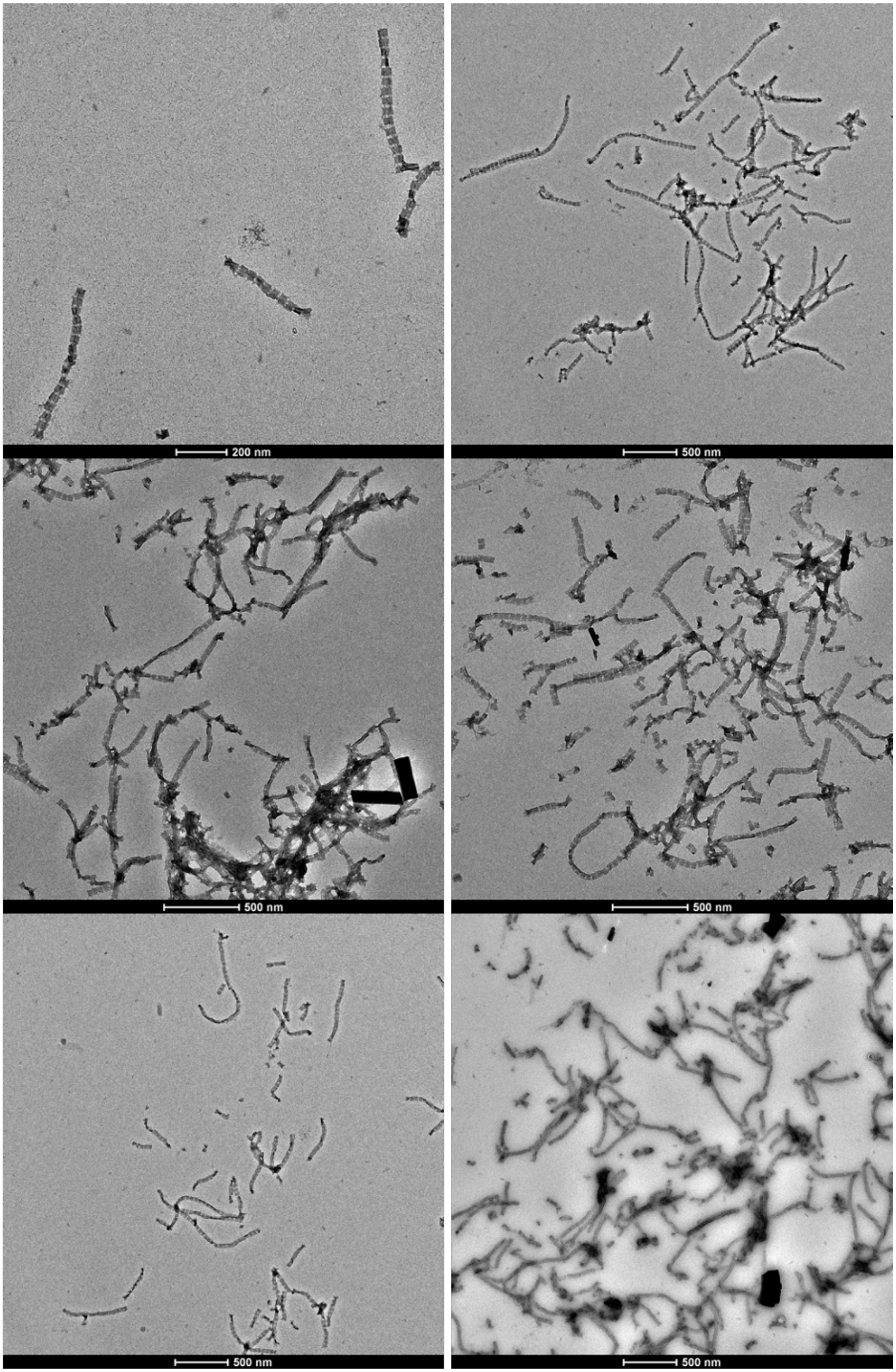

Figure S3. Representative TEM images of M_{64}^{20}.

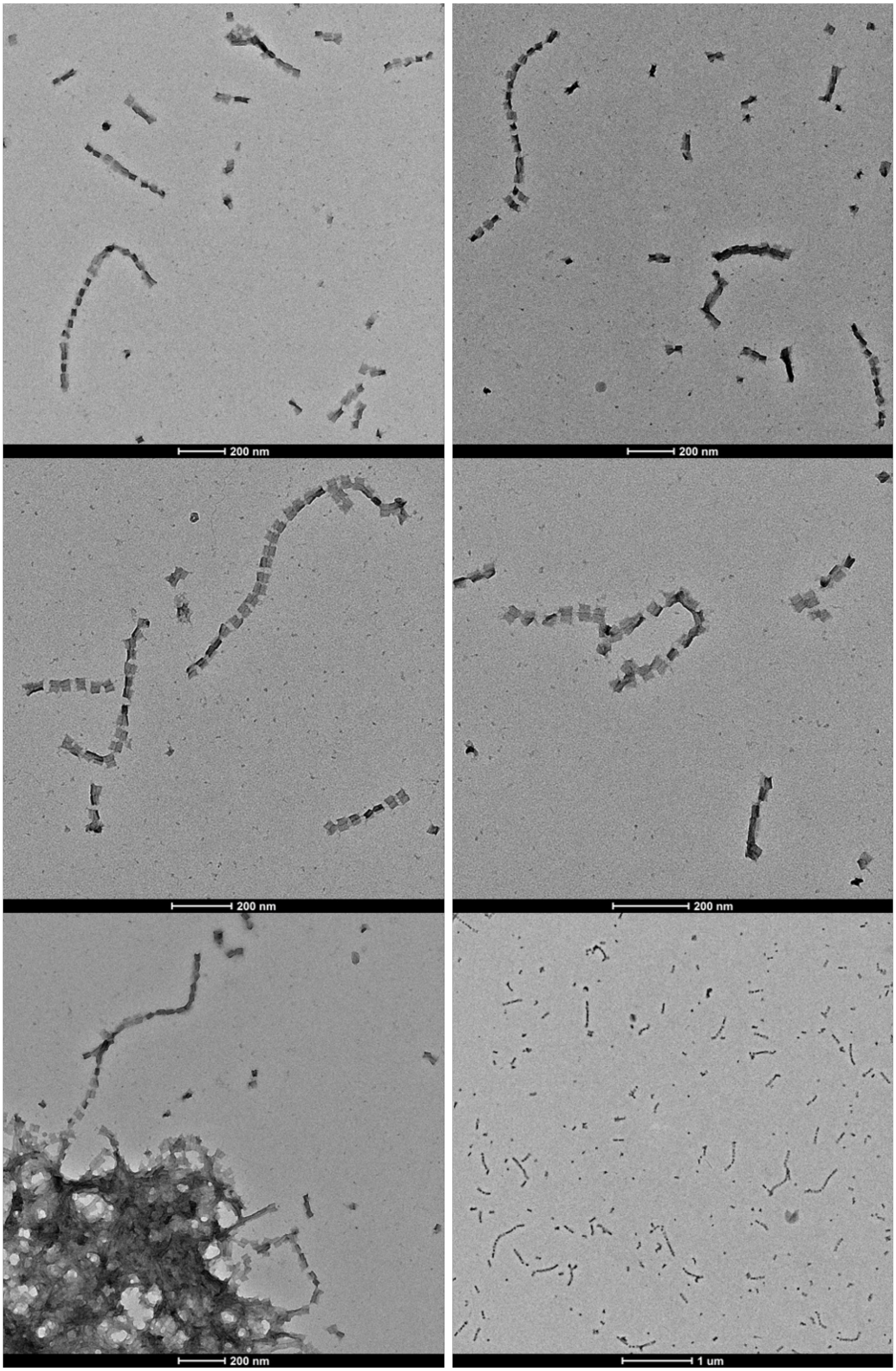

Figure S4. Representative TEM images of M_{16}^{40}.

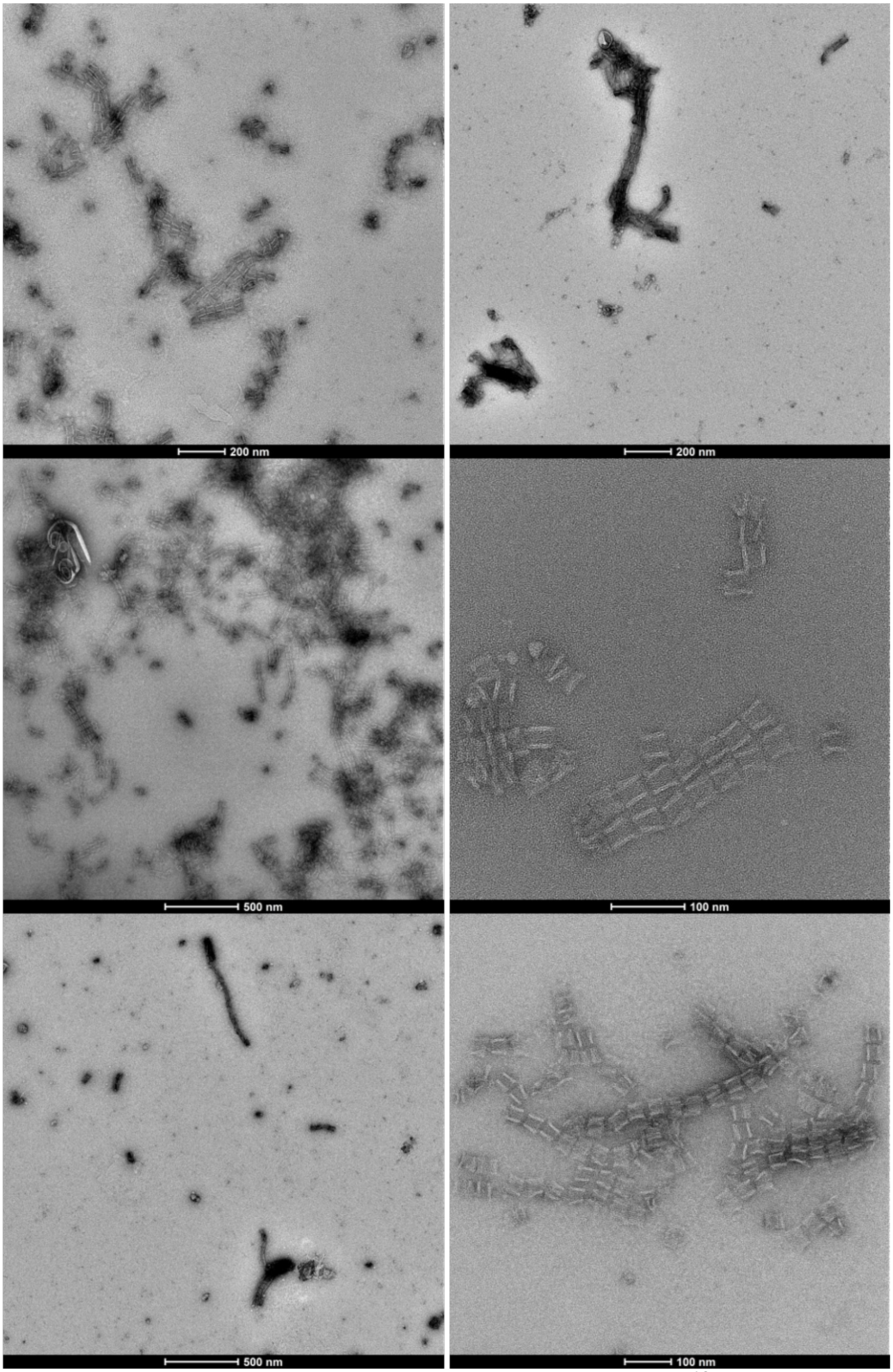

Figure S5. Representative TEM images of M_{32}^{40}.

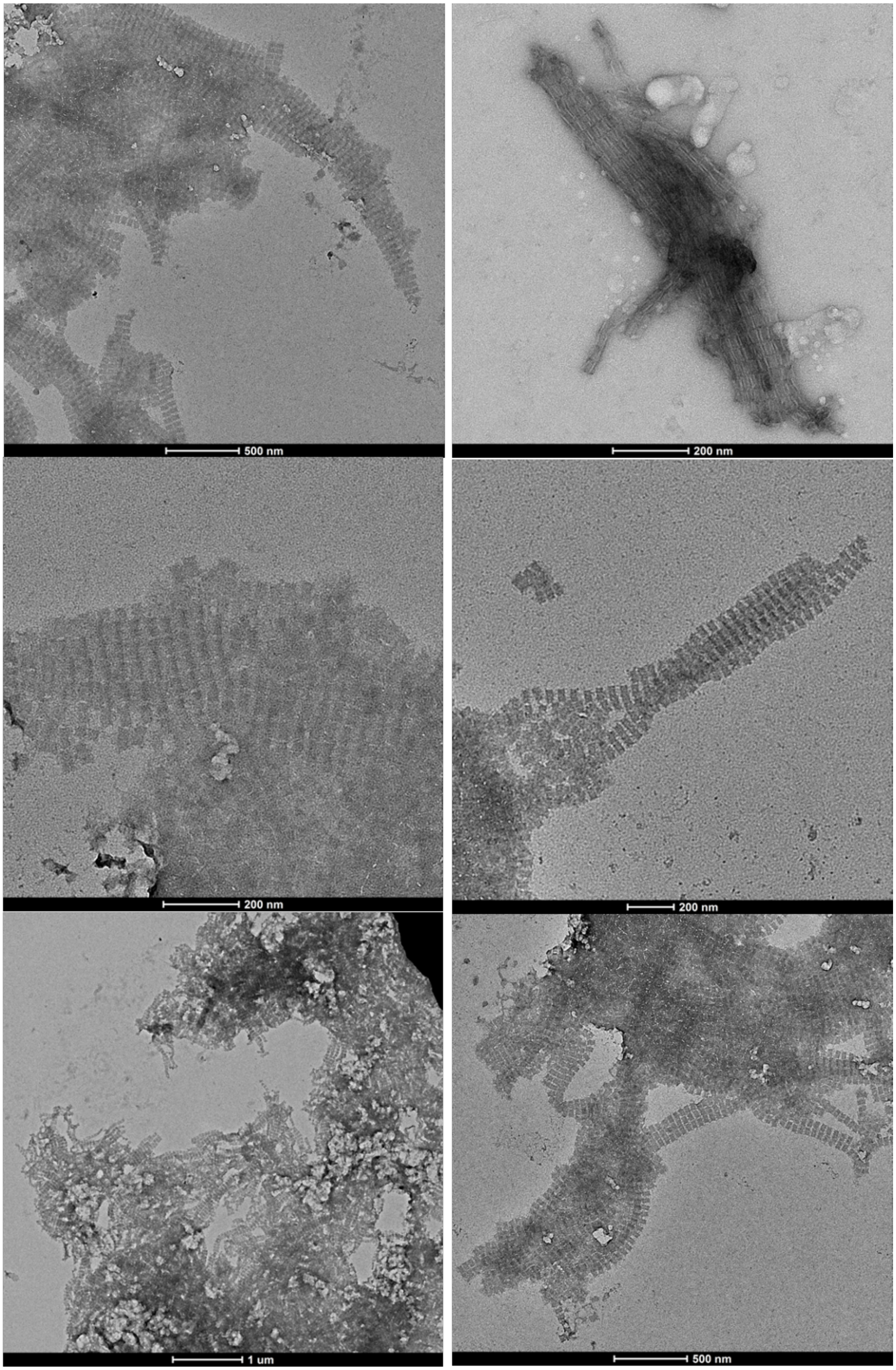

Figure S6. Representative TEM images of M_{64}^{40}.

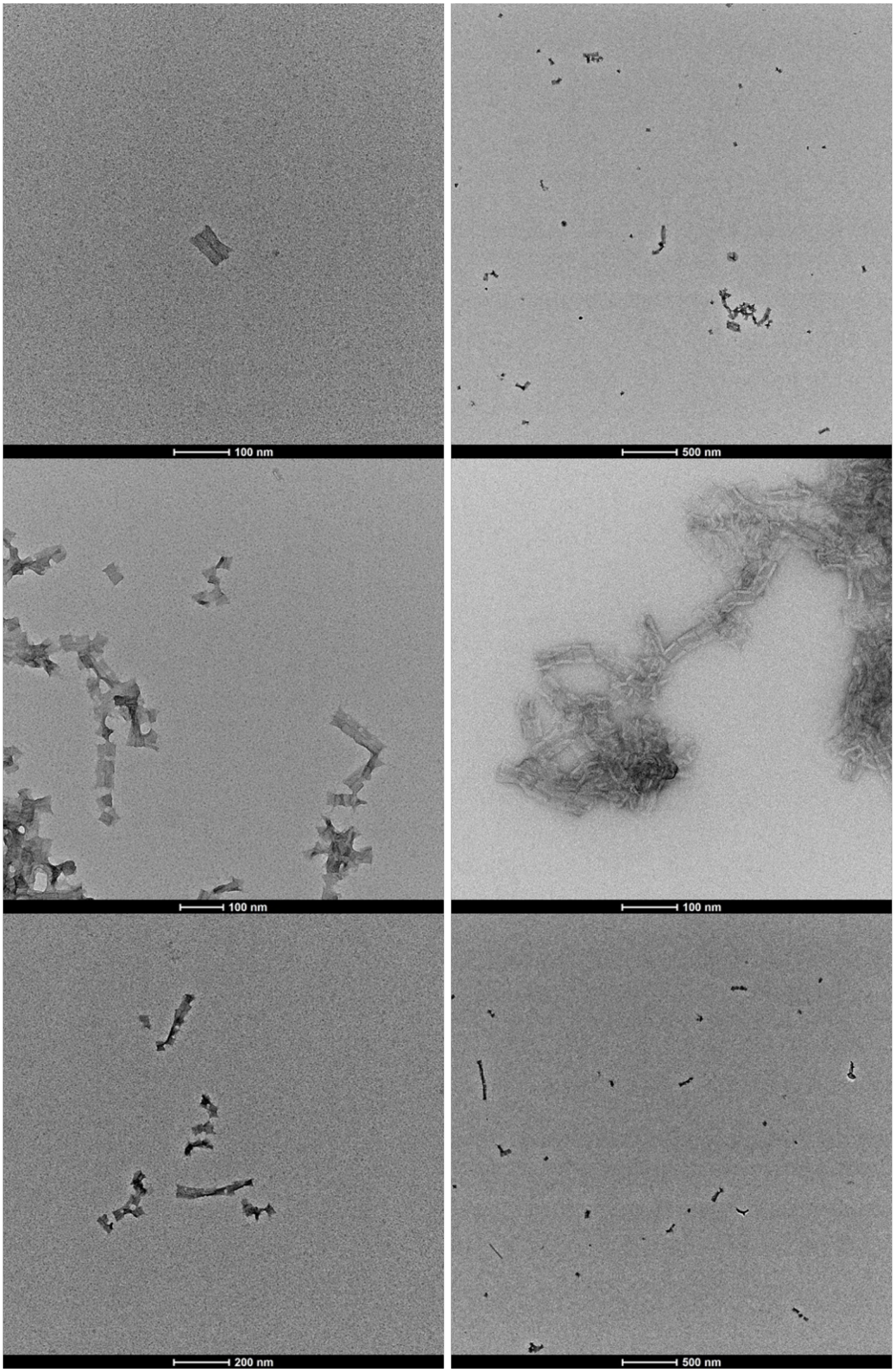

Figure S7. Representative TEM images of M_{32}^{10}.

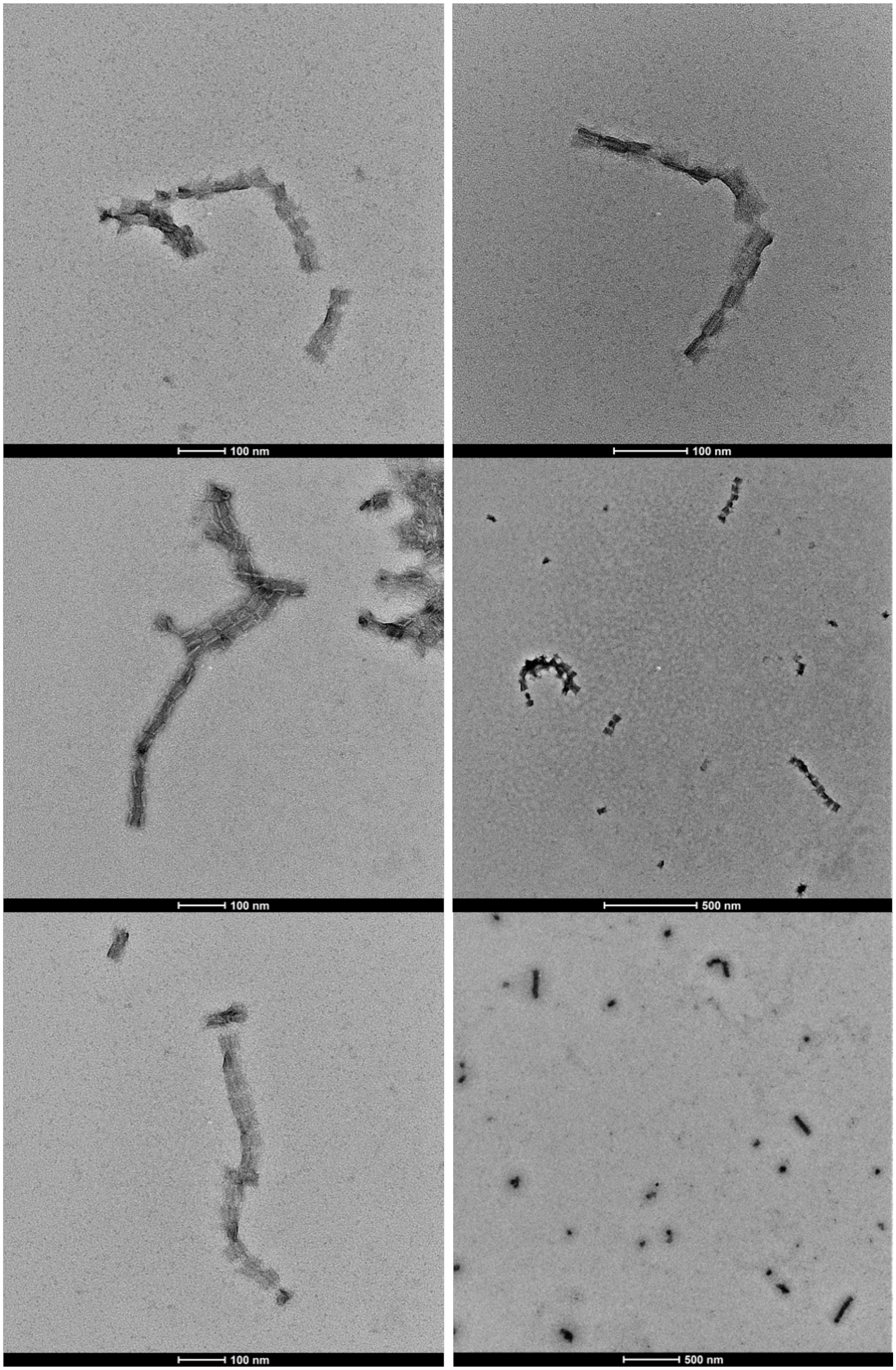

Figure S8. Representative TEM images of M_{32}^{30}.

Part 2. DNA Sequences

a. DNA Nano-Chamber

DNC is composed by a scaffold and 224 staples where 176 staples are kept unchanged. Only 48 staples which work on Z-direction connection are adjusted for tuning different connection properties. The 176 unchanged staples are listed as the following:

RS1	AAGCCAGAATGGCTGTTTAGTATCAAACAACGCCAACATGTAATTTGCATGGT
RS2	AAGCAAAAGTTTTAACGGGGAATCAATAAACTTCG
RS3	AAATATGCGCGACGCAGACGATTCAGTGAATACATTC
RS4	ATTCCACACCTGGGGTGTTGCGCTCACTGATGTTTT
RS5	AACCAACAGTTATGATACAGCTCAGAGCGCATTGACCCATCTTT
RS6	TGGCCAATTTTTGAACCCTAAAACATCG
RS7	ACGCTGCAATTTCATAAATCAAGGCGAATT
RS8	GAAAGGGGGGATCCCCGCATGCCTCGCTCACA
RS9	GTACGGTCAGACCTCATTA
RS10	TCATAATCATCGGCATAGCGTCAGAACGTCAC
RS11	GGCTGGCTAAAGTACAATGTTACTACCAACCTAAAA
RS12	AGTAATAAAAGGGAAGGACGCTCACGCTCATGG
RS13	AGGAGTCAGGATATGCTTTAAACAGTTCATTG
RS14	AGGCTAGCCGGAACGGCGGTCAGTATTAGCATCACCATAT
RS15	GCATTAACGCCACCCTCAGAGCCACCACCGAGT
RS16	TTTGGCGGGAGGTTTTGACGTATTGAGGGAGGG
RS17	GCTGTTTCTCAGTAGCGACAGAATC
RS18	ATTTCTTAAAGGAGCCTAATCTCCATTCAGCGGAG
RS19	AATCGAGGGGGTAATAGTAACAAAATAGAGAGCAACCAAAAGG
RS20	TGCAATTTTCAGTGCCTTGAGTAACAGTGATAAGTGCATTA
RS21	CGAACATTAAACGGGAGTTAAGCGAAAGGAGGTGA
RS22	GTCATATTAATTGCGCCTAATGCGGCCAAC
RS23	AAAGTATTAAAGAACGTTTTTTATACATCACG
RS24	TGAGGATCTACATGTATAAGCAAATATTCGCATTAATAGG
RS25	AACCTTAACGTTTCGTCACCAGATTACATTTAAAGAA
RS26	GAATGACGAGAAACACCAGCGTAACAATCAAGAGTGGCGCATA
RS27	AATGTTAGTTTGAAAGTACGTTGCTGAATATAATGCTAAG
RS28	GACTCCTTATTACGCAGCAAAAGGGCACCACGGGAGCCA
RS29	TATGGCGATCTAAAGACAGCCGGAACCCA
RS30	TGTAATACGTTGTACCTCATACAGGGTGGCAT
RS31	TAATATAAAACTAGCGTCTGGCGGCGGATATATTTTGTT
RS32	AGGCTGACCGTAATGGGATAGGAAC
RS33	CTTATTTGATAACTGCGAACGAGTAGATGTCA

RS34	AGAACCATGTTCAGCTATTTTTATTCTTTCCTTTTTAGCG
RS35	GTAGTCCGGCACCGCTTCTGTTCAGGCTCCTCTTC
RS36	CCTTACAGTACATTTGAATTGAAACAAA
RS37	TCTTCTTCAAAATCGCTTAGGTTAATTAATTTTC
RS38	ACCACCCTCGCCACCCCACCGTACGCTCAGTACCAG
RS39	CGCCTGTAGTAGAAAGGTTAGTAAAATCGGTTTATCACTTCTG
RS40	GGAAACTGATTGCCTTCACCAGGCGAAA
RS41	GAGGCTCCCTCAGCAAGGCCGCACAATGAC
RS42	CAGGGTGGAGAGGCGGAACCCCGCTTTCCAGTCG
RS43	GGCCTGGTGCGGGGCGCAACTTAACGCCA
RS44	TATACAAATTCTTACCAAAGAATAACGTGTGATGTTAATTTCA
RS45	TGAGAACATTCCACAGTTTTGTTCAGAGCC
RS46	AGCATTTTGCCACCCCTCAACGGCAAAATC
RS47	GAGAAGTCTGGATACCCCGGTACCCAAAGAAA
RS48	CCTTATATGGTGGTTGCCCCTGAGA
RS49	CTTACTCTCAATCATTGCTGAACCTC
RS50	TTTCCAGCAAGTTACAAAATTTATACAAACTACAA
RS51	GAGTGTCGCTATTGGGTTAT
RS52	AAATACCAGTAAAAGTTATTACATTTTGCA
RS53	TAAAGGATAACGTCAGATAAACAAAACGCGCAGATATATGTGAGTGAAT
RS54	CCCTCTTGCCTTTTTTCGGTCAGCAAAATCACC
RS55	ACGAGGTTTGAACCATATTTTATGCGT
RS56	ATAAGCTGAAAAGCAAGGCAAAGAATTAGATA
RS57	TTGCTTTGAATACACGAACAAC
RS58	AGTAAAGAAACGCAACAGCCTTTACAGAATTTTAA
RS59	AAAGTCCTAATTTGTTTTTGTTATCAAGAT
RS60	GCGCGGGGTTTTTCTTCTTCACCGCCTGGATCAA
RS61	ATTCATTTAGAAACAAAGTAACAGTAAAGAAATTGC
RS62	AACAACCAACGTTGAATTAATTGTTGAATTTTCTG
RS63	ATCCTGTTTGAAATCAAACAGTTTGGA
RS64	CAAATTAACATTGCAACACTATCGGACACGACC
RS65	AAAGAACGCAACCTCCGATAGGTCTTTAGATTAAG
RS66	AGCTAAATCGTTTTGCGGTTTTTAGAAGGGTGAGAAAGGCCGTGCC
RS67	GTGTAAAGCAACATACAGTGAATTCGTAATCATG
RS68	TAAAATCCAATCATCATAATTACTAGAAA
RS69	ACGTTGCCAGTTTGAGGTCGTCAGAGAGAGCAATAGCATAAAA
RS70	AAAAGAGAAGCCATATTCAACCGTTCTATTTT
RS71	CACTTGCCTGAGACAAGTCTGTCATCAGTGAGGCCACCGCAGA
RS72	AACGCCCTGACTAGACTTCAAATATCGCATGG
RS73	AGTATCACCGTCAAATTATTTTACCAGCGCC

RS74	CAAGCCGATGCAGAACCTAATTTAGTAATAAGAGA
RS75	AACCTCCCGACTTGGAAAGCAGCACCGTAAATCGT
RS76	AGCCACGAGCATGTAGAAACCAATCATTCACCCTC
RS77	ATAACTATATGTAAGTCGTCGAGAGGGTTTAGTGAATTTAGACC
RS78	ATTCGCCAGTGCCGGATAAGGACGACGACAGTATC
RS79	CGGCCAGTGCCAGTCACGCCACGCCAGCTGGC
RS80	AAATCCAAGATATAAGTATAGCCCGGAATAACACTGACGCCACCCCGTC
RS81	CTGGTCAGTTGGGCGGAACAAACTAATAGATTTAGAAGTATTAGAC
RS82	AAAATGAAAATAGAGACGACATTCAACCGCTTTCCA
RS83	TTGTTTGGATTATAGCGCATAACCGATATAGAAGGA
RS84	TTTACAAACAAACGTACGTAATGCCTAGGAGC
RS85	TTTTTTTTTTACCAGACGACGATAAAAACAATGTTTAGGTCTTTAAGAATGACCAGAC
RS86	TTTTTTTTTTGCAACAGCCTGTCGTGCCAGCTTTTTTTTTT
RS87	TTTTTTTTTTAACTTTCAACAGTAAAAAAAGTTTTTTTTTT
RS88	TTTTTTTTTTGACAGATGAACGGTGTACCAACTTTGAAAGAGTTTTTTTTTT
RS89	ACGCACTATCATAACCCTCGTTTTTTTTTTTTT
RS90	TTTTTTTTTTTTTTGCGGGTTTTAATTCGAGTTTTTTTTTT
RS91	AAAGATATGTTAGACATATAAAGCAGATAGCCGAACAAAGTTACCAGAAGTTTTTTTTTT
RS92	TTTTTTTTTTGGGCGATCCAGGAAGATCGCATTTTTTTTTT
RS93	TTTTTTTTTTCACCATCAATATGTTTATTTCTTTTTTTTTT
RS94	TTTTTTTTTTCAGATGATAAAATTATTTGCATTTTTTTTTT
RS95	TTTTTTTTTTCGTAAAACAGAAATACCTTTTTTTTTTTTTT
RS96	TTTTTTTTTTCAGGTCATGAGACAGTCAAATTTTTTTTTTT
RS97	TTTTTTTTTTACTCATCTTTGACCCCAAGAATACACTAAAACTTTTTTTTTT
RS98	TTTTTTTTTTCCAGAGCCACCACGAACCACCTTTTTTTTTT
RS99	TTTTTTTTTTAGAATTAACTGAACAAGAATTGAGTTAAGCCCTTTTTTTTTT
RS100	TTTTTTTTTTTGCATTAATGAATAGTGAGCTTTTTTTTTTT
RS101	TTTTTTTTTTGCGTTTTCAAAATCACCGGAATTTTTTTTTT
RS102	TTTTTTTTTTAATAATAAGAGCAAGAAACTTTCATCACGTGCATCTGGT
RS103	TTTTTTTTTTACGTTGGGAAGAAAAATCTAGTTGAGACATA
RS104	TTTTTTTTTTATTGTGTCGAAATCCGCGACCGAACTGACAG
RS105	TTTTTTTTTTACCTTTTTGAGAAAACTTTTTTTTTTTTTTT
RS106	TTTTTTTTTTATACATAACACAATCAATAGATTTTTTTTTT
RS107	TTTTTTTTTTCGTCTATCACCAGAATCCTGCAG
RS108	GATTTTGTATCATCGCCTGATAATTTTTTTTTT
RS109	ATCAGCTCATTATACCAGTCAGGTTTTTTTTTT
RS110	TTTTTTTTTTTTTTATCCCAATCCAAATAACGCATTAGACGGGTTTTTTTTTT
RS111	TTTTTTTTTTACCAGAGCCGCCGCCACGCCACCACGGAACCGCCT
RS112	TTTTTTTTTTTGCCTATTAGAAGGATTAGGATTTTTTTTTT
RS113	TTTTTTTTTTCGATTAAGTTGGGGTTGGGAATTTTTTTTTT

RS114	TTTTTTTTTTGCTCCAAAACAGCTTGATACCTTTTTTTTTT
RS115	TTTTTTTTTTGAAACCGAGGAAAGAAAATACTTTTTTTTTT
RS116	TTTTTTTTTTAAGGTAAAGTAATTTTATCAACAATAGATAAGTTTTTTTTTT
RS117	TTTTTTTTTTTTGATTCCCAATTGAGGTCATTTTTTTTTTT
RS118	TTTTTTTTTTACATAGCGATAGCGAGAGACTTTTTTTTTTT
RS119	TTTTTTTTTTTCCTGAACAAGAAAAATAAGTATTAAACCAAGTTTTTTTTTT
RS120	TTTTTTTTTTAACAATATTACCGCCAGCCCGTTGTAGCAATATTTTTTTTTT
RS121	TTTTTTTTTTGAAAGGAATTGAGTGCAACAGTGCCACGCTGATTTTTTTTTT
RS122	TTTTTTTTTTCTTCTTTGATTAGACGTCAAAGGGCGAAAAACTTTTTTTTTT
RS123	TTTTTTTTTTTTAGCGGGGTTTTCAGGAGGTTTTTTTTTT
RS124	TTTTTTTTTTGAGCCAGCAGCAAATGAAATTAAAAATACCGATTTTTTTTTT
RS125	TTTTTTTTTTTCAACTAATGCAGATATTTAGGAATACCACATTTTTTTTTT
RS126	ACCATATCGGCAATTCTCATATTCATCATTTTCAAATCAACAGTTTTTTTTTTTT
RS127	AAGTAGAGCCGCAGCGTTTGAGGAGGTTGAGAGGCAGAG
RS128	GAGCAGAGGGTAGAGAATAACTATCTTA
RS129	ATACAGAGATAAGAAGATAAAACAGAGGT
RS130	AGGAATCTAACGAGAGCCTTAATAACGTCA
RS131	CAAGAGAATCGTAGGTAAAATTAATGCTATGACCC
RS132	ACTAACAAGAAACCATTAAATCAATCCTGA
RS133	AATAGCGCAGTCTGAAACATGAAAATGCTGA
RS134	AGAGGCCACCCTCAGGCTTCTGT
RS135	TTAATCCAACAGAGCCCGAAATTATAGTGCATAGTACGAGA
RS136	CCAAATCTAAAACACCGCCGAAGGTTATCTAAAATAT
RS137	ACAAGAGTCCACAAGAAGCGGATTCCGAAATTTGGGCGC
RS138	AAGGTAAATCCATCGATTTAGAGCCATAGCCCCCTTAATAATCG
RS139	AAAGATTGTAGCTCTTTGCGTAAGCATAAA
RS140	CCGAAGCCAATAATTCGCATGTCAAAACGTTACGGAGAGG
RS141	AATTACGAGGCGATTTTAAAAC
RS142	GCGGATTTTCAGGGAAGGGTTCGCCTGA
RS143	GAGAGGACTGATAGTGGCTAT
RS144	AACGCCATCAAACTTTTTAAAGAACTGGCATGAGAGCGC
RS145	AATAATGTTATTGCGAATAATAATTT
RS146	GTAGCTATGCTGATAAGATTCAAAACCCTCATA
RS147	GAACGGTATCCCATCGCGCCTGTCTGTCCAGACG
RS148	TAGTTGCTTAAGAACGCGCCCAATGAGAACAAG
RS149	ATATGAGAATCGATACCGACACACCGGAGCAAGAC
RS150	AACATAGAAGAACTCAAAGGAAAAAATCGTCTGAAATGGATAGACA
RS151	ACAGGGAAGGAAACGATCCAGTTACCTGAATC
RS152	TTTCTCGCCCACTTGCTTTCACAGCATCGGATTCGACAA
RS153	AAAATTTAAATTGTATCATATGGCAAA

RS154	AAGTTTCAGGGCGCGACCTGTTTAGCATAA
RS155	CGGAAGCAAACTTGCTCCTGAGCTTAAGTGTCTGG
RS156	GCTATTTGAGTAATGTGATGAACGGAACC
RS157	AAACCTTCCTGTAGCCAGCAATGAAATATAACCCACACCCTGAAC
RS158	TATTTTGTAGGTGGCACAAACGTACGCAATAATAAC
RS159	CTCGTACCATTCGGTCGCTGAATCAAACCACGA
RS160	GCGGCCCGTATATATTATTCTCTGAATTTACC
RS161	CGAAACGGCTTGCAGGGTAAAAAGGGTAGCAACGGCTACA
RS162	AAATCTAATAGTGAGCCGGAAAAACGA
RS163	TAAGGGAACCTGCTCCACGGAGATATACCAAGCG
RS164	TATTTTACCAATAAAAAAAACATATACATTTCGCA
RS165	GGCGGTAGAAAGATTCATCACGTTAATAAGAACTGTTGTGAA
RS166	GAGTTGAGAAGTGGGACTCCATAATAACAT
RS167	GGGTTTTCCCAAGCTTGGGTACCGAGCTCAGCA
RS168	GTAGAATTATCAATCAATATCTTTGCCCGAAC
RS169	TAGTCTTTATACATTTTGCTTGCCCCTAACGG
RS170	TTACCAACGCATTACCGCGAGGCGTATCATTCCAA
RS171	TTACCTTATGACCTTCAAGCTGCTCGTCAATCA
RS172	CAATGAAAATTGACGGACCGACTTAATAAGTT
RS173	CATCAAGAGAATATACTAACGGATTAGAACCT
RS174	CAATTCTAATGCAACTACCATTAGTAGAGAGTACCT
RS175	TGTACCGTAGGTGTATTCAGAACCGCTGAGACTCCTCAAGTCGG
RS176	TTAACATAATGCAATGACGTTGTAAAGCGCC

b. Sequences for DNA Binding Strands

There are two types of "sticky ends": (i) staples working on lateral direction hybridization for nanofiber assembly and (ii) staples working on inner connection for AuNP location.

(1) For *k=64* design, insert desired poly-T sequences into [polyT] of the following strands:

Type A:

A32-1	GCGATTAC[polyT]TTATTCATTACCCAAATCAAAACGAGTATTTA
A32-2	AATCGGC[polyT]TTTTTTAATGGAAAGAATCCTTGAAAttttt
A32-3	GGTCCTAA[polyT]GCGTAAGAATACGTGGCACTATTTACATTGGC[polyT]CTCGGAAT
A32-4	CGTCGTAA[polyT]GAAATTGTTATCGCAGGTCG[polyT]TTACAGGC
A32-5	tttttACATCGGGCAATTACCTGAGC[polyT]GGAACTCT
A32-6	tttttAACGCAAGGCAAAATTAAGCA[polyT]TACAGGTC
A32-7	AACTCGGT[polyT]ACTCTAGAATGTGCTGCAAGGttttt
A32-8	GTATCAGC[polyT]TTTTTTTCTACAATTTTATCAAAATAAACAGCCATATTAttttt
A32-9	ATGACGCT[polyT]AAAAGAAGATGATACCTTTTTTTTT[polyT]ATACGCTG

A32-10	GCTTACAG[polyT]TAGCGTAAGATTTTGCTAAACttttt
A32-11	tttttACGAACCACCAGCGAACCCTTCTGACCTGAAAT[polyT]TTACGACG
A32-12	CCAGGATT[polyT]ATGGTTTAATTTCAACGTAAATTGGGCTTGAG[polyT]CAATCGG
A32-13	GACGACTT[polyT]GTGAATTAGCACCATTACCA[polyT]TCATAGGC
A32-14	TATCGGAC[polyT]TTTTTTCATTTGTTCCATATAACAGttttt
A32-15	AGTAGCCT[polyT]GAAGGCTTATCCGGTATTCATTTTGCACCCAG[polyT]TCGGAATC
A32-16	TTACGCAG[polyT]TTTTTTTAGATTCACCAGTCCCTTGCTGGTAATATCCAGttttt
A32-17	tttttAAATTCATATGGTCATTAAAG[polyT]GTATCACG
A32-18	TATCACGG[polyT]ATAAAGCCTCAGAGCTATA[polyT]TTTTTTATTGACGC
A32-19	CCGAAGTT[polyT]AGCAAGCCCAATACTCATAG[polyT]GTTCGCAA
A32-20	tttttTTTAGTACCATTTTCAGGGA[polyT]ACTACTGG
A32-21	GCCTAAGT[polyT]AGCAAGGCCGGAACTGTAGCttttt
A32-22	tttttTACCGCACTCATCAGCAAGCAAATCAGATATAT[polyT]CATACGGT
A32-23	tttttAACTCACAGCTGTTTCCTGTG[polyT]ATCATCGG
A32-24	ACCAAATCTTGACAAGAACCGGA[polyT]TACAGCGT
A32-25	TCCATAGG[polyT]GAGTAACATTCTGATTATttttt
A32-26	tttttCAAATATATTTTAAAATAAGG[polyT]ATCCGAGT
A32-27	GGAATTGATAATTTAACCAAATTTTTGTTAAATCAGTTTT[polyT]GTCCAGAT
A32-28	GCACTTAG[polyT]CTCATTTTCAGAAAAGCCCCA[polyT]TACTGGCA
A32-29	GCAGTCAT[polyT]ATCGTAACACATTAAATGTGA[polyT]GATGTCAC
A32-30	TCTGCGAA[polyT]AAAAATCAGACTGGATAGCG[polyT]AGGCTTCA
A32-31	TACGCGAT[polyT]CGTTAAATGTATAAAGCCAAC[polyT]ATGTCCAG
A32-32	GTAGTCCA[polyT]GATCGTCATTGAGGACTAAAG[polyT]CAAGTTGC
A32-33	GTTCAATAAATCGATTGGCCTTGATATTTTTT[polyT]GCTCATAG
A32-34	tttttCTCCAGCCAGCTTATGGGCGCTT[polyT]CAAGTTCG
A32-35	GTCCGATA[polyT]TTTTTCACAAACACAGTAAGCGTCA[polyT]TACTGGAC
A32-36	AGTAGTCC[polyT]AAAACAGGAAGATAAGGCTATttttt
A32-37	TACTGACG[polyT]CCAATACTGCGGAATCGTCATAAATATTCAGAA
A32-38	TAGTAGCC[polyT]TTGCCCCAGCAGTGAGACGGttttt
A32-39	GAATCCGT[polyT]GGGTTGAGCGGTCCACGCTGG[polyT]CTATGAGC
A32-40	tttttCTTCAAAGCGAACCATAAATCT[polyT]CAGCAGTT
A32-41	TGGCTACA[polyT]ACTTTTTCATGAGGAAGTTTCAGAGGCAACAGC
A32-42	tttttGATAGTTGCGCCGTTTTGCGG[polyT]ACAGTTCG
A32-43	CACGATGT[polyT]GCGAGTAACAACCCGTCGGATTCTCCGTGGGTC
A32-44	GTTACATTTGAGGATTAGAGCCGTCAAT[polyT]CTAATCGG
A32-45	TCGGCATA[polyT]ACATGGCTTTTGAATGCCCCCttttt
A32-46	CAAGTGTTGTTCAGAATAGCCCGAGATA[polyT]GCTCTAAG
A32-47	AATGTCCG[polyT]TTTTTAGATAATATTAATTTTAAAAG[polyT]ACTTGGAC
A32-48	GACCTTGA[polyT]GCTCAACAGTAGGGCTTAATTAAAGTACCGACAAttttt

Type B:

B32-1	TTACGACG[polyT]ATTCATTACCCAAATCAAAACGAGTATTTA
B32-2	GTAATCGC[polyT]TTTTTAATGGAAAGAATCCTTGAAAttttt
B32-3	GTCCGATA[polyT]GCGTAAGAATACGTGGCACTATTTACATTGGC[polyT]GACCTGTA
B32-4	AAGTCGTC[polyT]GAAATTGTTATCGCAGGTCGT[polyT]GCCTATGA
B32-5	tttttACATCGGGCAATTACCTGAGC[polyT]CCGATTGA
B32-6	tttttAACGCAAGGCAAAATTAAGCA[polyT]ACCGTATG
B32-7	ACTTAGGC[polyT]ACTCTAGAATGTGCTGCAAGGttttt
B32-8	AACTTCGG[polyT]TTTTTCTACAATTTTATCAAAATAAACAGCCATATTAttttt
B32-9	AATCCTGG[polyT]AAAAGAAGATGATACCTTTTTTTT[polyT]ACGCTGTA
B32-10	CTGCGTAA[polyT]AGCGTAAGATTTTGCTAAACttttt
B32-11	tttttACGAACCACCAGCGAACCCTTCTGACCTGAAA[polyT]GCGTCAAT
B32-12	ACCGAGTT[polyT]ATGGTTTAATTTCAACGTAAATTGGGCTTGAG[polyT]GCCTGTAA
B32-13	AGCCGATT[polyT]GTGAATTAGCACCATTACCA[polyT]AGAGTTCC
B32-14	GCTGATAC[polyT]TTTTTCATTTGTTCCATATAACAGttttt
B32-15	CTGTAAGC[polyT]GAAGGCTTATCCGGTATTCATTTTGCACCCAG[polyT]CCAGTAGT
B32-16	CCGTGATA[polyT]TTTTTTTAGATTCACCAGTCCCTTGCTGGTAATATCCAGttttt
B32-17	tttttAAATTCATATGGTCATTAAAG[polyT]CAGCGTAT
B32-18	AGGCTACT[polyT]ATAAAGCCTCAGAGCTATA[polyT]GATTCCGA
B32-19	TTAGGACC[polyT]AGCAAGCCCAATACTCATAG[polyT]ATTCCGAG
B32-20	tttttTTTAGTACCATTTTCAGGGA[polyT]CGTCGTAA
B32-21	AGCGTCAT[polyT]AGCAAGGCCGGAACTGTAGCttttt
B32-22	tttttTACCGCACTCATCAGCAAGCAAATCAGATATA[polyT]TTGCGAAC
B32-23	tttttAACTCACAGCTGTTTCCTGTG[polyT]CGTGATAC
B32-24	ACCAAATCTTGACAAGAACCGGA[polyT]CCGATGAT
B32-25	TTCGCAGA[polyT]GAGTAACATTCTGATTATttttt
B32-26	tttttCAAATATATTTTAAAATAAGG[polyT]CGAACTGT
B32-27	GGAATTGATAATTTAACCAAATTTTTGTTAAATCAG[polyT]CTGGACAT
B32-28	ATCGCGTA[polyT]CTCATTTTCAGAAAAGCCCCA[polyT]ACTCGGAT
B32-29	TATGCCGA[polyT]ATCGTAACACATTAAATGTGA[polyT]GTCCAGTA
B32-30	ATGACTGC[polyT]AAAAATCAGACTGGATAGCG[polyT]CGAACTTG
B32-31	TGTAGCCA[polyT]CGTTAAATGTATAAAGCCAAC[polyT]GCAACTTG
B32-32	ACGGATTC[polyT]GATCGTCATTGAGGACTAAAG[polyT]CTTAGAGC
B32-33	GTTCAATAAATCGATTGGCCTTGATA[polyT]GTCCAAGT
B32-34	tttttCTCCAGCCAGCTTATGGGCGC[polyT]CTATGAGC
B32-35	CGGACATT[polyT]CACAAACACAGTAAGCGTCA[polyT]CCGATTAG
B32-36	TCAAGGTC[polyT]AAAACAGGAAGATAAGGCTATttttt
B32-37	ACATCGTG[polyT]CCAATACTGCGGAATCGTCATAAATATTCAGAA
B32-38	CTAAGTGC[polyT]GCCCCAGCAGTGAGACGGttttt
B32-39	GGACTACT[polyT]TTTTGGGTTGAGCGGTCCACGCTGG[polyT]GCCAGTA

B32-40	tttttCTTCAAAGCGAACCATAAATCTTTT[polyT]GTGACATC
B32-41	GGCTACTA[polyT]TACTTTTTCATGAGGAAGTTTCAGAGGCAACAGC
B32-42	tttttGATAGTTGCGCCGTTTTGCGG[polyT]GCTCATAG
B32-43	TATCGGAC[polyT]GCGAGTAACAACCCGTCGGATTCTCCGTGGGTC
B32-44	GTTACATTTGAGGATTAGAGCCGTCAA[polyT]AACTGCTG
B32-45	CCTATGGA[polyT]ACATGGCTTTTGAATGCCCCCttttt
B32-46	CAAGTGTTGTTCAGAATAGCCCGAGATA[polyT]ATCTGGAC
B32-47	CGTCAGTA[polyT]TTTTAGATAATATTAATTTTAAAAG[polyT]TGAAGCCT
B32-48	TGGACTAC[polyT]GCTCAACAGTAGGGCTTAATTAAAGTACCGACAAttttt

(2) For *k=32* design, insert desired poly-T sequences into [polyT] of the following strands:

Type A:

A16-1	tttttTTATTCATTACCCAAATCAAAACGAGTATTTA
A16-2	tttttTTTTTTAATGGAAAGAATCCTTGAAAttttt
A16-3	tttttGCGTAAGAATACGTGGCACTATTTACATTGGCttttt
A16-4	tttttGAAATTGTTATCGCAGGTCGttttt
A16-5	tttttACATCGGGCAATTACCTGAGCttttt
A16-6	tttttAACGCAAGGCAAAATTAAGCAttttt
A16-7	tttttACTCTAGAATGTGCTGCAAGGttttt
A16-8	TTTTTTTCTACAATTTTATCAAAATAAACAGCCATATTAttttt
A16-9	tttttAAAAGAAGATGATACCTTTTTTTTT
A16-10	tttttTAGCGTAAGATTTTGCTAAACttttt
A16-11	tttttACGAACCACCAGCGAACCCTTCTGACCTGAAATttttt
A16-12	tttttATGGTTTAATTTCAACGTAAATTGGGCTTGAGttttt
A16-13	tttttGTGAATTAGCACCATTACCAttttt
A16-14	TTTTTTCATTTGTTCCATATAACAGttttt
A16-15	tttttGAAGGCTTATCCGGTATTCATTTTGCACCCAGttttt
A16-16	TTTTTTTAGATTCACCAGTCCCTTGCTGGTAATATCCAGttttt
A16-17	tttttAAATTCATATGGTCATTAAAGttttt
A16-18	tttttATAAAGCCTCAGAGCTATAttttt
A16-19	tttttAGCAAGCCCAATACTCATAGttttt
A16-20	tttttTTTAGTACCATTTTCAGGGAttttt
A16-21	tttttAGCAAGGCCGGAACTGTAGCttttt
A16-22	tttttTACCGCACTCATCAGCAAGCAAATCAGATATATttttt
A16-23	tttttAACTCACAGCTGTTTCCTGTGttttt
A16-24	ACCAAATCTTGACAAGAACCGGAttttt
A16-25	TCCATAGG[polyT]GAGTAACATTCTGATTATttttt
A16-26	tttttCAAATATATTTTAAAATAAGG[polyT]ATCCGAGT
A16-27	GGAATTGATAATTTAACCAAATTTTTGTTAAATCAGTTTT[polyT]GTCCAGAT

A16-28	GCACTTAG[polyT]CTCATTTTCAGAAAAGCCCCA[polyT]TACTGGCA
A16-29	GCAGTCAT[polyT]ATCGTAACACATTAAATGTGA[polyT]GATGTCAC
A16-30	TCTGCGAA[polyT]AAAAATCAGACTGGATAGCG[polyT]AGGCTTCA
A16-31	TACGCGAT[polyT]CGTTAAATGTATAAAGCCAAC[polyT]ATGTCCAG
A16-32	GTAGTCCA[polyT]GATCGTCATTGAGGACTAAAG[polyT]CAAGTTGC
A16-33	GTTCAATAAATCGATTGGCCTTGATATTTTTT[polyT]GCTCATAG
A16-34	tttttCTCCAGCCAGCTTATGGGCGCTT[polyT]CAAGTTCG
A16-35	GTCCGATA[polyT]TTTTTCACAAACACAGTAAGCGTCA[polyT]TACTGGAC
A16-36	AGTAGTCC[polyT]AAAACAGGAAGATAAGGCTATttttt
A16-37	TACTGACG[polyT]CCAATACTGCGGAATCGTCATAAATATTCAGAA
A16-38	TAGTAGCC[polyT]TTGCCCCAGCAGTGAGACGGttttt
A16-39	GAATCCGT[polyT]GGGTTGAGCGGTCCACGCTGG[polyT]CTATGAGC
A16-40	tttttCTTCAAAGCGAACCATAAATCT[polyT]CAGCAGTT
A16-41	TGGCTACA[polyT]ACTTTTTCATGAGGAAGTTTCAGAGGCAACAGC
A16-42	tttttGATAGTTGCGCCGTTTTGCGG[polyT]ACAGTTCG
A16-43	CACGATGT[polyT]GCGAGTAACAACCCGTCGGATTCTCCGTGGGTC
A16-44	GTTACATTTGAGGATTAGAGCCGTCAAT[polyT]CTAATCGG
A16-45	TCGGCATA[polyT]ACATGGCTTTTGAATGCCCCCttttt
A16-46	CAAGTGTTGTTCAGAATAGCCCGAGATA[polyT]GCTCTAAG
A16-47	AATGTCCG[polyT]TTTTTAGATAATATTAATTTTAAAAG[polyT]ACTTGGAC
A16-48	GACCTTGA[polyT]GCTCAACAGTAGGGCTTAATTAAAGTACCGACAAttttt

Type B:

B16-1	tttttATTCATTACCCAAATCAAAACGAGTATTTA
B16-2	tttttTTTTTAATGGAAAGAATCCTTGAAAttttt
B16-3	tttttGCGTAAGAATACGTGGCACTATTTACATTGGCttttt
B16-4	tttttGAAATTGTTATCGCAGGTCGTttttt
B16-5	tttttACATCGGGCAATTACCTGAGCttttt
B16-6	tttttAACGCAAGGCAAAATTAAGCAttttt
B16-7	tttttACTCTAGAATGTGCTGCAAGGttttt
B16-8	TTTTTCTACAATTTTATCAAAATAAACAGCCATATTAttttt
B16-9	tttttAAAAGAAGATGATACCTTTTTTTT
B16-10	tttttAGCGTAAGATTTTGCTAAACttttt
B16-11	tttttACGAACCACCAGCGAACCCTTCTGACCTGAAAtttttt
B16-12	tttttATGGTTTAATTTCAACGTAAATTGGGCTTGAGtttttt
B16-13	tttttGTGAATTAGCACCATTACCAttttt
B16-14	tttttTTTTTCATTTGTTCCATATAACAGttttt
B16-15	tttttGAAGGCTTATCCGGTATTCATTTTGCACCCAGttttt
B16-16	TTTTTTTAGATTCACCAGTCCCTTGCTGGTAATATCCAGttttt

B16-17 tttttAAATTCATATGGTCATTAAAGttttt
B16-18 tttttATAAAGCCTCAGAGCTATAttttt
B16-19 tttttAGCAAGCCCAATACTCATAGttttt
B16-20 tttttTTTAGTACCATTTTCAGGGAttttt
B16-21 tttttAGCAAGGCCGGAACTGTAGCttttt
B16-22 tttttTACCGCACTCATCAGCAAGCAAATCAGATATAttttt
B16-23 tttttAACTCACAGCTGTTTCCTGTGttttt
B16-24 ACCAAATCTTGACAAGAACCGGAttttt
B16-25 TTCGCAGA[polyT]GAGTAACATTCTGATTATttttt
B16-26 tttttCAAATATATTTTAAAATAAGG[polyT]CGAACTGT
B16-27 GGAATTGATAATTTAACCAAATTTTTGTTAAATCAG[polyT]CTGGACAT
B16-28 ATCGCGTA[polyT]CTCATTTTCAGAAAAGCCCCA[polyT]ACTCGGAT
B16-29 TATGCCGA[polyT]ATCGTAACACATTAAATGTGA[polyT]GTCCAGTA
B16-30 ATGACTGC[polyT]AAAAATCAGACTGGATAGCG[polyT]CGAACTTG
B16-31 TGTAGCCA[polyT]CGTTAAATGTATAAAGCCAAC[polyT]GCAACTTG
B16-32 ACGGATTC[polyT]GATCGTCATTGAGGACTAAAG[polyT]CTTAGAGC
B16-33 GTTCAATAAATCGATTGGCCTTGATA[polyT]GTCCAAGT
B16-34 tttttCTCCAGCCAGCTTATGGGCGC[polyT]CTATGAGC
B16-35 CGGACATT[polyT]CACAAACACAGTAAGCGTCA[polyT]CCGATTAG
B16-36 TCAAGGTC[polyT]AAAACAGGAAGATAAGGCTATttttt
B16-37 ACATCGTG[polyT]CCAATACTGCGGAATCGTCATAAATATTCAGAA
B16-38 CTAAGTGC[polyT]GCCCCAGCAGTGAGACGGttttt
B16-39 GGACTACT[polyT]TTTTGGGTTGAGCGGTCCACGCTGG[polyT]GCCAGTA
B16-40 tttttCTTCAAAGCGAACCATAAATCTTTT[polyT]GTGACATC
B16-41 GGCTACTA[polyT]TACTTTTTCATGAGGAAGTTTCAGAGGCAACAGC
B16-42 tttttGATAGTTGCGCCGTTTTGCGG[polyT]GCTCATAG
B16-43 TATCGGAC[polyT]GCGAGTAACAACCCGTCGGATTCTCCGTGGGTC
B16-44 GTTACATTTGAGGATTAGAGCCGTCAA[polyT]AACTGCTG
B16-45 CCTATGGA[polyT]ACATGGCTTTTGAATGCCCCCttttt
B16-46 CAAGTGTTGTTCAGAATAGCCCGAGATA[polyT]ATCTGGAC
B16-47 CGTCAGTA[polyT]TTTTAGATAATATTAATTTTAAAAG[polyT]TGAAGCCT
B16-48 TGGACTAC[polyT]GCTCAACAGTAGGGCTTAATTAAAGTACCGACAAttttt

(3) For *k=16* design, insert desired poly-T sequences into [polyT] of the following strands:

Type A:

A8-1 tttttTTATTCATTACCCAAATCAAAACGAGTATTTA
A8-2 TTTTTTAATGGAAAGAATCCTTGAAAttttt
A8-3 tttttGCGTAAGAATACGTGGCACTATTTACATTGGCttttt
A8-4 tttttGAAATTGTTATCGCAGGTCGttttt

A8-5	ttttACATCGGGCAATTACCTGAGCtttt
A8-6	ttttAACGCAAGGCAAAATTAAGCAtttt
A8-7	ttttACTCTAGAATGTGCTGCAAGGtttt
A8-8	ttttTTTTTTTCTACAATTTTATCAAAATAAACAGCCATATTAtttt
A8-9	ATGACGCT[polyT]AAAAGAAGATGATACCTTTTTTTTT[polyT]ATACGCTG
A8-10	ttttTAGCGTAAGATTTTGCTAAACtttt
A8-11	ttttACGAACCACCAGCGAACCCTTCTGACCTGAAAT[polyT]TTACGACG
A8-12	ttttATGGTTTAATTTCAACGTAAATTGGGCTTGAGtttt
A8-13	ttttGTGAATTAGCACCATTACCAtttt
A8-14	TTTTTTCATTTGTTCCATATAACAGtttt
A8-15	AGTAGCCT[polyT]GAAGGCTTATCCGGTATTCATTTTGCACCCAG[polyT]TCGGAATC
A8-16	TTTTTTTAGATTCACCAGTCCCTTGCTGGTAATATCCAGtttt
A8-17	ttttAAATTCATATGGTCATTAAAG[polyT]GTATCACG
A8-18	TATCACGG[polyT]ATAAAGCCTCAGAGCTATA[polyT]TTTTTTATTGACGC
A8-19	ttttAGCAAGCCCAATACTCATAGtttt
A8-20	ttttTTTAGTACCATTTTCAGGGA[polyT]ACTACTGG
A8-21	ttttAGCAAGGCCGGAACTGTAGCtttt
A8-22	ttttTACCGCACTCATCAGCAAGCAAATCAGATATATtttt
A8-23	ttttAACTCACAGCTGTTTCCTGTG[polyT]ATCATCGG
A8-24	ACCAAATCTTGACAAGAACCGGA[polyT]TACAGCGT
A8-25	ttttGAGTAACATTCTGATTATtttt
A8-26	ttttCAAATATATTTTAAAATAAGGtttt
A8-27	GGAATTGATAATTTAACCAAATTTTTGTTAAATCAGTTTT[polyT]GTCCAGAT
A8-28	ttttCTCATTTTCAGAAAAGCCCCAtttt
A8-29	GCAGTCAT[polyT]ATCGTAACACATTAAATGTGA[polyT]GATGTCAC
A8-30	ttttAAAAATCAGACTGGATAGCGtttt
A8-31	TACGCGAT[polyT]CGTTAAATGTATAAAGCCAAC[polyT]ATGTCCAG
A8-32	GTAGTCCA[polyT]GATCGTCATTGAGGACTAAAG[polyT]CAAGTTGC
A8-33	GTTCAATAAATCGATTGGCCTTGATATTTTTT[
A8-34	ttttCTCCAGCCAGCTTATGGGCGCTTtttt
A8-35	GTCCGATA[polyT]TTTTTCACAAACACAGTAAGCGTCA[polyT]TACTGGAC
A8-36	ttttAAAACAGGAAGATAAGGCTATtttt
A8-37	ttttCCAATACTGCGGAATCGTCATAAATATTCAGAA
A8-38	ttttTTGCCCCAGCAGTGAGACGGtttt
A8-39	ttttGGGTTGAGCGGTCCACGCTGGtttt
A8-40	ttttCTTCAAAGCGAACCATAAATCT[polyT]CAGCAGTT
A8-41	ttttACTTTTTCATGAGGAAGTTTCAGAGGCAACAGC
A8-42	ttttGATAGTTGCGCCGTTTTGCGGtttt
A8-43	ttttGCGAGTAACAACCCGTCGGATTCTCCGTGGGTC

A8-44 GTTACATTTGAGGATTAGAGCCGTCAAT[polyT]CTAATCGG
A8-45 ttttACATGGCTTTTGAATGCCCCCtttt
A8-46 CAAGTGTTGTTCAGAATAGCCCGAGATA[polyT]GCTCTAAG
A8-47 TTTTTAGATAATATTAATTTTAAAAGtttt
A8-48 ttttGCTCAACAGTAGGGCTTAATTAAAGTACCGACAAtttt

Type B:

B8-1 ttttATTCATTACCCAAATCAAAACGAGTATTTA
B8-2 TTTTTAATGGAAAGAATCCTTGAAAtttt
B8-3 ttttGCGTAAGAATACGTGGCACTATTTACATTGGCtttt
B8-4 ttttGAAATTGTTATCGCAGGTCGTtttt
B8-5 ttttACATCGGGCAATTACCTGAGCtttt
B8-6 ttttAACGCAAGGCAAAATTAAGCAtttt
B8-7 ttttACTCTAGAATGTGCTGCAAGGtttt
B8-8 TTTTTCTACAATTTTATCAAAATAAACAGCCATATTAtttt
B8-9 AATCCTGG[polyT]AAAAGAAGATGATACCTTTTTTTT[polyT]ACGCTGTA
B8-10 ttttAGCGTAAGATTTTGCTAAACtttt
B8-11 ttttACGAACCACCAGCGAACCCTTCTGACCTGAAA[polyT]GCGTCAAT
B8-12 ttttATGGTTTAATTTCAACGTAAATTGGGCTTGAGtttt
B8-13 ttttGTGAATTAGCACCATTACCAtttt
B8-14 TTTTTCATTTGTTCCATATAACAGtttt
B8-15 CTGTAAGC[polyT]GAAGGCTTATCCGGTATTCATTTTGCACCCAG[polyT]CCAGTAGT
B8-16 TTTTTTTAGATTCACCAGTCCCTTGCTGGTAATATCCAGtttt
B8-17 ttttAAATTCATATGGTCATTAAAG[polyT]CAGCGTAT
B8-18 AGGCTACT[polyT]ATAAAGCCTCAGAGCTATA[polyT]GATTCCGA
B8-19 ttttAGCAAGCCCAATACTCATAGtttt
B8-20 ttttTTTAGTACCATTTTCAGGGA[polyT]CGTCGTAA
B8-21 ttttAGCAAGGCCGGAACTGTAGCtttt
B8-22 ttttTACCGCACTCATCAGCAAGCAAATCAGATATAtttt
B8-23 ttttAACTCACAGCTGTTTCCTGTG[polyT]CGTGATAC
B8-24 ACCAAATCTTGACAAGAACCGGA[polyT]CCGATGAT
B8-25 ttttGAGTAACATTCTGATTATtttt
B8-26 ttttCAAATATATTTTAAAATAAGGtttt
B8-27 GGAATTGATAATTTAACCAAATTTTTGTTAAATCAG[polyT]CTGGACAT
B8-28 ttttCTCATTTTCAGAAAAGCCCCAtttt
B8-29 TATGCCGA[polyT]ATCGTAACACATTAAATGTGA[polyT]GTCCAGTA
B8-30 ttttAAAAATCAGACTGGATAGCGtttt
B8-31 TGTAGCCA[polyT]CGTTAAATGTATAAAGCCAAC[polyT]GCAACTTG
B8-32 ACGGATTC[polyT]GATCGTCATTGAGGACTAAAG[polyT]CTTAGAGC

B8-33	GTTCAATAAATCGATTGGCCTTGATAttttt
B8-34	tttttCTCCAGCCAGCTTATGGGCGCttttt
B8-35	CGGACATT[polyT]CACAAACACAGTAAGCGTCA[polyT]CCGATTAG
B8-36	tttttAAAACAGGAAGATAAGGCTATttttt
B8-37	tttttCCAATACTGCGGAATCGTCATAAATATTCAGAA
B8-38	tttttGCCCCAGCAGTGAGACGGttttt
B8-39	tttttTTTTGGGTTGAGCGGTCCACGCTGGttttt
B8-40	tttttCTTCAAAGCGAACCATAAATCTTTT[polyT]GTGACATC
B8-41	tttttTACTTTTTCATGAGGAAGTTTCAGAGGCAACAGC
B8-42	tttttGATAGTTGCGCCGTTTTGCGGttttt
B8-43	tttttGCGAGTAACAACCCGTCGGATTCTCCGTGGGTC
B8-44	GTTACATTTGAGGATTAGAGCCGTCAA[polyT]AACTGCTG
B8-45	tttttACATGGCTTTTGAATGCCCCCttttt
B8-46	CAAGTGTTGTTCAGAATAGCCCGAGATA[polyT]ATCTGGAC
B8-47	tttttTTTTAGATAATATTAATTTTAAAAGttttt
B8-48	tttttGCTCAACAGTAGGGCTTAATTAAAGTACCGACAAttttt

For anchoring AuNP into DNCs, the DNA strand grafted to AuNP surface is: GCCTCCTACCTATTTTTT/3ThioMC3-D/

To endow DNC with the ability of AuNP anchoring, use the following strands to replace the strands RS169-RS176 (marked in blue) in Part 3a:

RS169*	TAGGTAGGATTTTTTTTTTTTAGTCTTTATACATTTTGCTTGCCCCTAACGG
RS170*	TAGGTAGGATTTTTTTTTTTTTACCAACGCATTACCGCGAGGCGTATCATTCCAA
RS171*	TAGGTAGGATTTTTTTTTTTTTACCTTATGACCTTCAAGCTGCTCGTCAATCA
RS172*	TAGGTAGGATTTTTTTTTTTCAATGAAAATTGACGGACCGACTTAATAAGTT
RS173*	TAGGTAGGATTTTTTTTTTTCATCAAGAGAATATACTAACGGATTAGAACCT
RS174*	TAGGTAGGATTTTTTTTTTTCAATTCTAATGCAACTACCATTAGTAGAGAGTACCT
RS175*	TAGGTAGGATTTTTTTTTTTTGTACCGTAGGTGTATTCAGAACCGCTGAGACTCCTCAAGTCGG
RS176*	TAGGTAGGATTTTTTTTTTTTTAACATAATGCAATGACGTTGTAAAGCGCC

Part 3. Simulation of Nano-Monomer Using Molecular Dynamics

a. Raspberry Model and Steric Interactions

All Molecular Dynamics (MD) simulations we performed are in the ESPResSo software package.[1] The effects of the background fluid were handled implicitly, *via* the Langevin thermostat[2] at a fixed temperature T (LD). In LD simulations, stochastic translational and rotational equations of motion are numerically integrated over time t:

$$M_i \frac{d\vec{v}_i}{dt} = \vec{\mathrm{F}}_i - \Gamma_{Tl}\vec{v}_i + 2\vec{\xi}_i^{Tl}, \quad \textit{Eq. 1}$$

$$I_i \frac{d\vec{\omega}_i}{dt} = \vec{\tau}_i - \Gamma_R\vec{\omega}_i + 2\vec{\xi}_i^{R}, \quad \textit{Eq. 2}$$

where for the i-th particle in Eq. 1, M_i is the mass tensor, $\vec{v}_i$ denotes the translational velocity, $\vec{\mathrm{F}}_i$ is the force acting on it, Γ_{Tl} denotes the translational friction coefficient, $\vec{\xi}_i^{Tl}$ is a stochastic force modelling the random forces of the implicit solvent. In Eq. 2, I_i denotes i-th particle inertia tensor, $\vec{\omega}_i$ is its rotational velocity, $\vec{\tau}_i$ is torque acting on it, Γ_R denotes the rotational friction coefficient, and the $\vec{\xi}_i^{R}$ is a stochastic torque serving for the same purpose as $\vec{\xi}_i^{Tl}$. Both stochastic terms satisfy the following conditions on their time averages:[3]

$$\langle \vec{\xi}^{R/Tl} \rangle_t = 0\,; \quad \textit{Eq. 3}$$

$$\langle \vec{\xi}_l^{Tl/R}(t)\vec{\xi}_k^{Tl/R}(t') \rangle = 2\Gamma_{Tl/R}k_B T\delta_{l,k}\delta(t-t'); \quad \textit{Eq. 4}$$

where $k, l = x, y, z$. Both forces and torques in Eq. 1 and Eq. 2 are calculated from interparticle interaction potentials. We used full periodic boundary conditions, and for the integration, the velocity Verlet algorithm.[4]

For an ensemble of spherical particles, to address the effective excluded volume, the typical approach to model their interactions in MD is to use Weeks-Chandler-Andersen pair potential (WCA):[5]

$$U_{WCA}(r) = \begin{cases} U_{LJ}(r) - U_{LJ}(r_{cut}), & r < r_{cut} \\ 0, & r \geq r_{cut} \end{cases}$$

Eq. 5

where and $U_{LJ}(r)$ is the conventional Lennard-Jones potential:

$$U_{LJ}(r) = 4\epsilon\{(\sigma/r)^{12} - (\sigma/r)^{6}\}$$

Eq. 6

where σ is the characteristic diameter of the sphere and the cutoff value is $r_{cut} = 2^{1/6}\sigma$. Parameter ϵ defines the energy scale of the repulsion. For non-spherical particles, or spherical particles whose surface properties need to be taken in to account, there are two ways of modelling steric interactions. One could solve an algebraic system of equations on each MD integration step to check for the overlap of complex shapes; Alternatively, one can construct complex shapes out of spherical particles – WCA centers of required size σ. We use the latter method which, albeit less accurate, is very flexible, can be applied to arbitrary shapes and is computationally efficient. Realizations of monomers with complex shape using this method are usually addressed as a raspberry particles.[6] Therefore, we refer to representations of DNA nano-chambers here as raspberry cubes. We successfully used such cubic raspberry particles earlier, for investigating magnetic and charged cubes.[7-10] Positions, radii and parameters for the steric interactions between the WCA-spheres that make out raspberry cubes are calculated using the superball model for $q = 2$, developed by,[7] where q is the shape parameter in the superball equation given by:

$$|\frac{x}{r}|^{2q} + |\frac{y}{r}|^{2q} + |\frac{z}{r}|^{2q} \leq 1$$

Eq. 7

At $\lim_{q\to\infty} f(q)$, where $f(q)$ is Eq. 7, $f(q)$ is describing a cube with perfectly sharp edges. With $q = 1$, Eq. 7 describes a sphere with radius r centered at $r = 0$.

Regarding computational efficiency and raspberry particles, we make use of virtual sites, a concept implemented in ESPResSo. Virtual sites are massless particles, excluded from equations of motion (see Eq. 1 and Eq. 2) calculations. They are fixed with respect to the reference frame of an otherwise conventional, non-virtual particle, that is propagated based on integrations of the equations of motion. Therefore, any group of virtual sites must be assigned to a reference, conventional particle. It is possible to define any number of virtual sites in arbitrary arrangements with respect to the reference frame of a reference particle they are assigned to. The interactions between virtual-virtual and virtual-real particles can be specified using any suitable potential. All forces exerted on the virtual sites are instantaneously propagated to the reference particle. These features allow to define rigid bodies with any shape by defining proper arrangements of virtual sites.

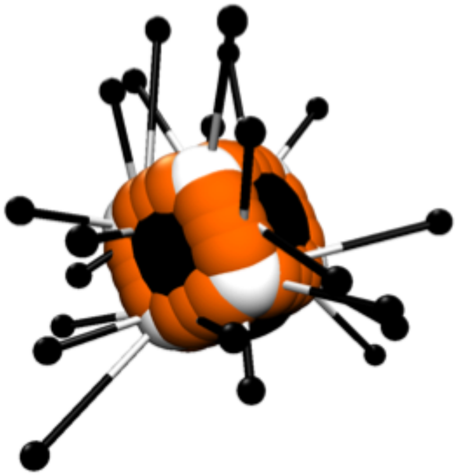

Figure S9. Raspberry particle model of DNA nano-monomers, with sticky linkers arranged to correspond M_{32}^{l} nano-monomers design (small black spheres). Central reference particle is shown as large black sphere. Virtual sites outlining the DNC vertices shown in orange. Virtual sites populating the DNC edges shown in white.

b. Simulating Self-Assembly of Monomers

In this work, we refrain from using explicit representations of DNA binding strands, such as a freely jointed chain representation,[10] or more sophisticated approaches such as oxDNA.[11] Such models are rather costly in terms of computational resources. Regardless, our reasoning for using simpler representations of binding strands, is that we wanted to keep the models as general as possible while still representative of the phenomenology we are interested in reproducing. We model DNA binding strands as finitely extendable springs, described $FENE$ potential:[12]

$$U_{FENE}(r) = \frac{-K_f r_f^2}{2} \ln\left\{1 - \left(\frac{r - r_0}{r_f}\right)^2\right\} \qquad \textit{Eq. 8}$$

where K_f is the rigidity of the bond, r_f is the maximal stretching length and r_0 is the equilibrium bond length of a FENE bond, corresponding to spacer length $\boldsymbol{l}$ of the experimental system. We attach DNA binding strands to raspberry cubes to form nano-monomers with three different bond arrangements, closely resembling the placement of binding strands on M_{16}^{l}, M_{32}^{l} and M_{64}^{l} nano-monomers (with length $\boldsymbol{l}$ and number of bonds $\boldsymbol{k}$ as M_k^l). In Figure S9 one can find a visualization of a raspberry cube nano-monomer employed in this study. Just as in the experimental setup, in our simulations we distinguish two complementary types of nano-monomers, A and B. We model the sticky ends of the binding strands as massless, attractive LJ-spheres Eq. 6 with diameter $\sigma_{sticky} = 0.05$, $\epsilon = 10$, with the cutoff value is $r_{cut} = 2.5\sigma_{sticky}$. We calculate r_0 of the binding strands, shown in black in Figure S9, as $\alpha(\sigma_{virtual} + \sigma_{sticky})$ which is an α multiple of the touching, center-to-center distance (minimum of the steric repulsion potential) between the raspberry DNC particle to which the binding strand is anchored to (WCA diameter $\sigma_{virtual}$), and the sticky end. Choosing M_k^l and ϵ allows us to finely tune the monomeric reactivity.

We reproduce how "differentiated polychromatic bonds"[1] facilitate face-specific bonding, by having sticky ends on nano-monomers A attractive to sticky ends on nano-monomers B. To dynamically form bonds between bonding strands, a collision detection procedure is employed. It is based on a distance criterion: if two sticky ends appear within a given distance one from another (σ_{sticky}), we permanently bond them via rigid FENE bonds where we choose $K_f = 100$, $r_0 = 2^{1/6}\sigma_{sticky}$, and $r_f = 2r_0$. Once a pair of sticky ends is bonded, they become inert to the rest of the system except trough excluded volume interactions. No other particle can be bonded to them after this point. In this way we emulate bond valence.

We run twelve parallel LD simulations, at $k_bT = 1$, each of which containing 40 randomly placed raspberry nano-monomers with binding strands $\alpha = 2$, corresponding to M_k^{40} nano-monomers, in a fully periodic simulation box ($12\sigma \times 12\sigma \times 12\sigma$). Timestep used in the equations of motion Eq. 1 and Eq. 2 is 10^{-2}. We firstly make sure that system settles into a configuration, where there are no overlapping particles. We achieve this by generating the initial raspberry cubes nano-monomer configuration by placing them randomly on a uniform grid, and subsequently

ruining 1000 integrations with high $\Gamma_{Tl} = \Gamma_R = 100$. For the rest of the simulation time, we reduce friction to $\Gamma_{Tl} = \Gamma_R = 1$. We simulate fast cooling by running an annealing procedure, where we decrease the thermostat temperature from $T = 1$ to $T = 0$ in 10 steps, each step being 5000 integrations long. During the annealing, we run a collision detection procedure described above. We monitor sticky ends present in the simulation box.

References

1. Weik, F.; Weeber, R.; Szuttor, K.; Breitsprecher, K.; de Graaf, J.; Kuron, M.; Landsgesell, J.; Menke, H.; Sean, D.; Holm, C. *The European Physical Journal Special Topics* **2019,** 227, (14), 1789-1816.
2. Allen, M. P.; Tildesley, D. J., *Computer Simulation of Liquids: Second Edition*. 2 ed.; Oxford University Press: Oxford, 2017; p 640.
3. Uhlenbeck, G. E.; Ornstein, L. S. *Physical Review* **1930,** 36, (5), 823-841.
4. Rapaport, D. C., *The Art of Molecular Dynamics Simulation*. 2 ed.; Cambridge University Press: Cambridge, 2004.
5. Weeks, J. D.; Chandler, D.; Andersen, H. C. *The Journal of Chemical Physics* **1971,** 54, (12), 5237-5247.
6. Lobaskin, V.; Dünweg, B. *New Journal of Physics* **2004,** 6, 54-54.
7. Donaldson, J. G.; Linse, P.; Kantorovich, S. S. *Nanoscale* **2017,** 9, (19), 6448-6462.
8. Donaldson, J. G.; Kantorovich, S. S. *Nanoscale* **2015,** 7, (7), 3217-28.
9. Rosenberg, M.; Dekker, F.; Donaldson, J. G.; Philipse, A. P.; Kantorovich, S. S. *Soft Matter* **2020,** 16, (18), 4451-4461.
10. Vargas-Lara, F.; Starr, F. W.; Douglas, J. F. *Soft Matter* **2017,** 13, (44), 8309-8330.
11. Ouldridge, T. E.; Louis, A. A.; Doye, J. P. *Phys Rev Lett* **2010,** 104, (17), 178101.
12. Meirovitch, H.; Livne, S. *The Journal of Chemical Physics* **1988,** 88, (7), 4507-4515.
